# The ultimate legs of Chilopoda (Myriapoda): a review on their morphological disparity and functional variability

**DOI:** 10.7717/peerj.4023

**Published:** 2017-11-14

**Authors:** Matthes Kenning, Carsten H.G. Müller, Andy Sombke

**Affiliations:** 1 Zoological Institute and Museum, Cytology and Evolutionary Biology, Ernst-Moritz-Arndt Universität Greifswald, Greifswald, Germany; 2 Zoological Institute and Museum, General and Systematic Zoology, Ernst-Moritz-Arndt Universität Greifswald, Greifswald, Germany

**Keywords:** Arthropodium, Behavior, Centipedes, Disparity, Evolution, Modification, Morphology, Transformation

## Abstract

The arthropodium is the key innovation of arthropods. Its various modifications are the outcome of multiple evolutionary transformations, and the foundation of nearly endless functional possibilities. In contrast to hexapods, crustaceans, and even chelicerates, the spectrum of evolutionary transformations of myriapod arthropodia is insufficiently documented and rarely scrutinized. Among Myriapoda, Chilopoda (centipedes) are characterized by their venomous forcipules—evolutionarily transformed walking legs of the first trunk segment. In addition, the posterior end of the centipedes’ body, in particular the ultimate legs, exhibits a remarkable morphological heterogeneity. Not participating in locomotion, they hold a vast functional diversity. In many centipede species, elongation and annulation in combination with an augmentation of sensory structures indicates a functional shift towards a sensory appendage. In other species, thickening, widening and reinforcement with a multitude of cuticular protuberances and glandular systems suggests a role in both attack and defense. Moreover, sexual dimorphic characteristics indicate that centipede ultimate legs play a pivotal role in intraspecific communication, mate finding and courtship behavior. We address ambiguous identifications and designations of podomeres in order to point out controversial aspects of homology and homonymy. We provide a broad summary of descriptions, illustrations, ideas and observations published in past 160 years, and propose that studying centipede ultimate legs is not only essential in itself for filling gaps of knowledge in descriptive morphology, but also provides an opportunity to explore diverse pathways of leg transformations within Myriapoda.

## Introduction

### Arthropod legs

The arthropodium can be regarded as one, if not *the* eponymous key innovation of arthropods. In taking on a sheer plethora of functions (locomotion/propulsion, food capture, handling and ingestion, communication and copulation, respiration, and reception of various stimuli), arthropodia are among the most versatile, most specialized, and hence, most widely modified features known in arthropods. The enormous disparity of morphologies has been regarded as a key component of the evolution of arthropods (Boxshall, 2013). Despite the increasing reliability and robustness of molecular approaches, the morphology of arthropodia, as well as aspects on their development still play a major role in arthropod phylogeny (Boxshall, 1997; Kukalová-Peck, 1997; Walossek & Müller, 1997; Bitsch, 2001; Klass & Kristensen, 2001; Wolf & Harzsch, 2002).

Although morphological disparity and functional diversity are by no means restricted to a particular segment, there are regions on the arthropod body where this disparity becomes especially evident, such as the head, which is equipped with various mouthparts and sensory appendages, or the terminal region with the gonopods. Head appendages are commonly considered the outcome of multiple and independent transformations of former walking legs (e.g., Waloszek et al., 2005). Comparable modifications comprise the convergent transformation of the anterior-most thoracic arthropodia which resulted in at least one pair of accessory mouthparts. These so-called maxillipedes are commonly found in all Chilopoda (Myriapoda), as well as in many in-groups of Crustacea (e.g., see reviews by McLaughlin, 1982; Schram, 1986), in which they are considered diagnostic, if not apomorphic characters.

As the name suggests, arthropodia are subdivided into distinct podomeres, also termed ‘segments’ and ‘annuli’, which in turn can be reduced or fused to a varying degree. According to Snodgrass (1935) and Boxshall (2004), true leg segments are defined by the presence of intrinsic muscles that originate, insert or attach to these segments whereas annuli lack intrinsic muscles. However, an unambiguous identification is not always as straightforward as this approach suggests. Transformation of arthropodia mostly implies changes in the number and proportion of podomeres, as well as the addition or modification of trichomes, sensilla or other sclerotized protuberances. Most functional and morphological modifications of stenopodial arthropodia are widely known from hexapods, but also crustaceans, and are prominent examples in scientific and public textbooks. In the course of arthropod evolution, walking legs have been transformed into raptorial legs (e.g., Stomatopoda, Mantodea, Mantispidae), grasping legs (e.g., Branchiopoda), digging legs (e.g., Gryllotalpidae, larvae of Cicadoidea), cleaning legs (e.g., Anomala), waving legs (e.g., Ucidae), swimming legs (e.g., Corixidae, Dytiscidae, Portunidae), jumping legs (e.g., Orthoptera), sensory legs (Protura), or collecting legs (e.g., Hymenoptera) just to name a few (McLaughlin, 1982; Dathe, 2003). Body appendages are thus promising objects to study the processes of adaptive evolutionary transformations.

### Centipede legs

**Figure 1:**
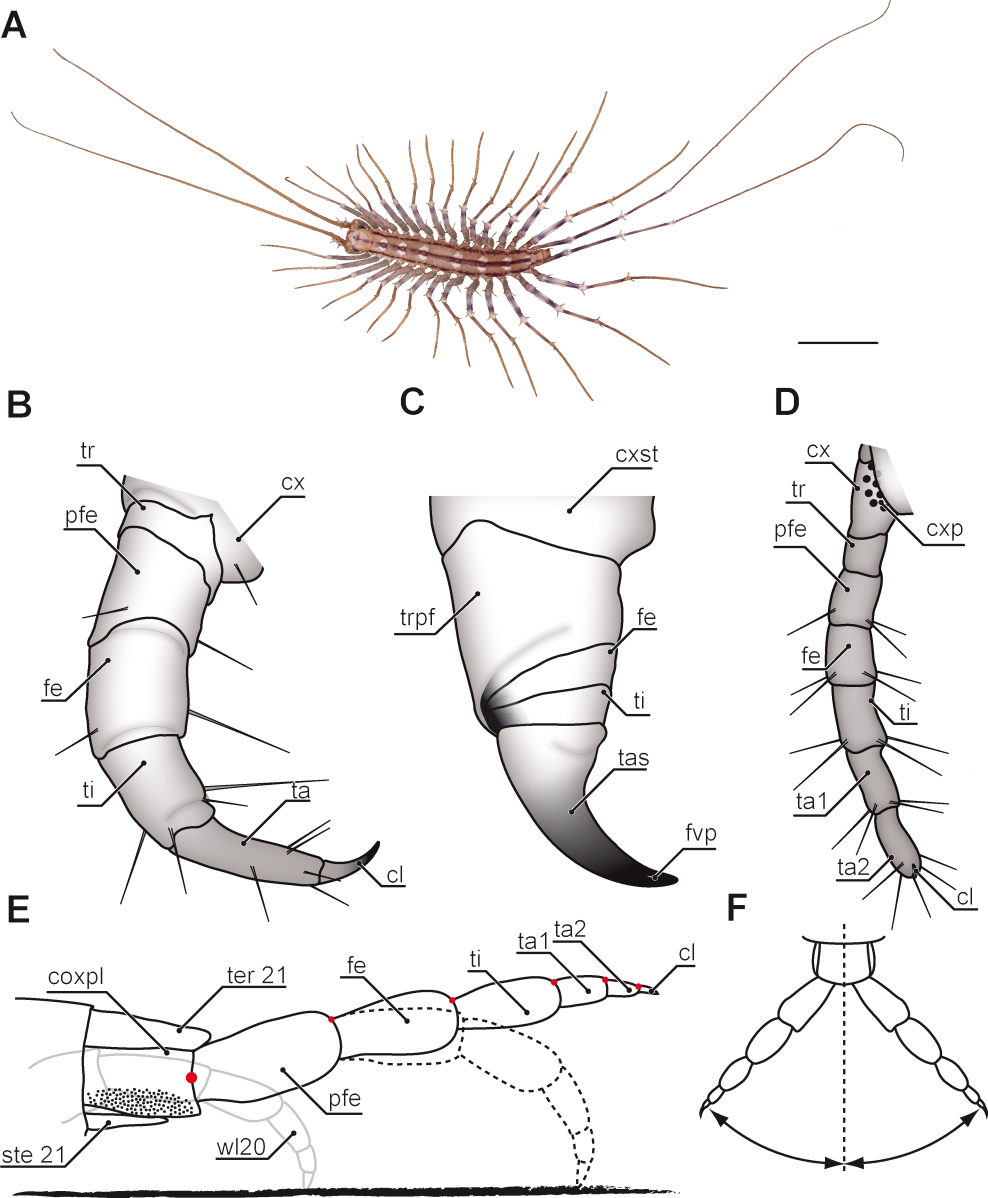
Overview of centipede appendages. (A) Habitus of *Scutigera coleoptrata* from dorsal. Walking legs gradually increasing in length along body axis. Note the resemblance of anterior (left) and posterior (right) pole of the body (Original). (B–D) Schematic representations of serially homologous, modified arthropodia of *Geophilus flavus*, not to scale. (B) Walking leg 10 (view from posterior, Original). (C) The forcipule with the typical shared joint of distal podomeres (view from ventral, modified after Haug et al., 2014). (D) The ultimate leg with coxal pores (view from ventral, Original). (E) Articulation and movement of ultimate legs in *Scolopendra morsitans* (lateral view, modified after Jangi, 1961). Elevated leg (solid line), resting leg (dotted line), and walking leg 20 for size comparison (solid grey line). Dorsoventral movements are restricted by dorsally located pivot joints (red dots). (F) Horizontal movements are restricted by the joint between coxopleura and prefemur (compare E, modified after Jangi, 1961). Scale bar: A 1 cm. Abbreviations: cl, pretarsal claw; cx, coxa: cxp, coxal pores; cxpl, coxopleura; cxst, coxosternite; fe, femur; fvp, forcipular venom pore; pfe, prefemur; ste21, sternum 21; ta, tarsus; ta1, tarsus 1; ta2, tarsus 2; tas, tarsungulum; ter21, tergite 21; ti, tibia; tr, trochanter; trpf, trochanteroprefemur; wl20, walking leg 20.

**Figure 2:**
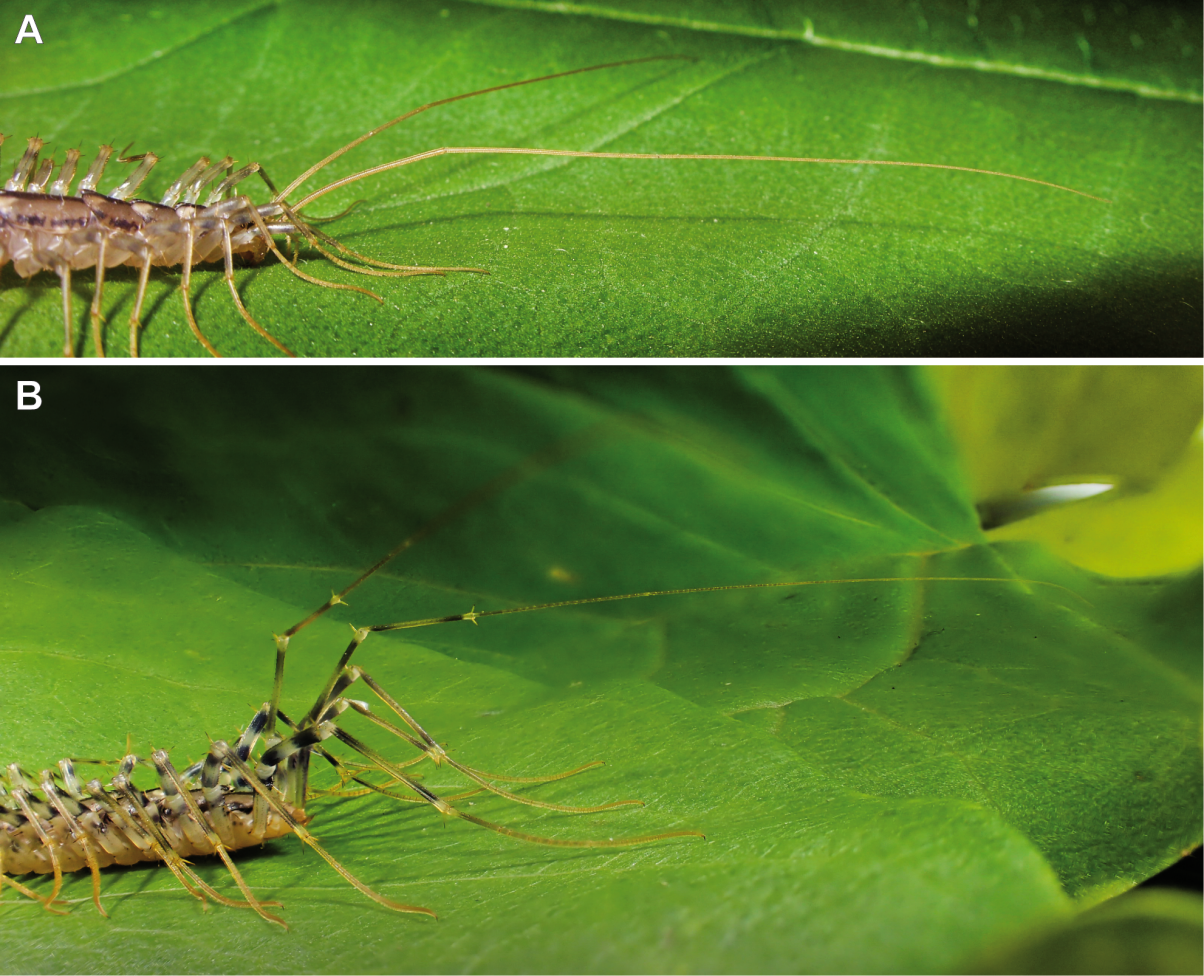
Posture of antennae and ultimate legs in Scutigeromorpha. (A) Anterior body of *Scutigera coleoptrata*. Note the typical position of antennae with the proximal part directed slightly upward and the distal part (divided by the first antennal node) held parallel to the substrate (Original). (B) Posterior body of *S. coleoptrata*. Walking leg tarsi and pretarsal claws are in contact with the substrate. Ultimate leg prefemora and femora are directed upward, tibiae and tarsalia are positioned in parallel to the substrate (Original).

**Figure 3:**
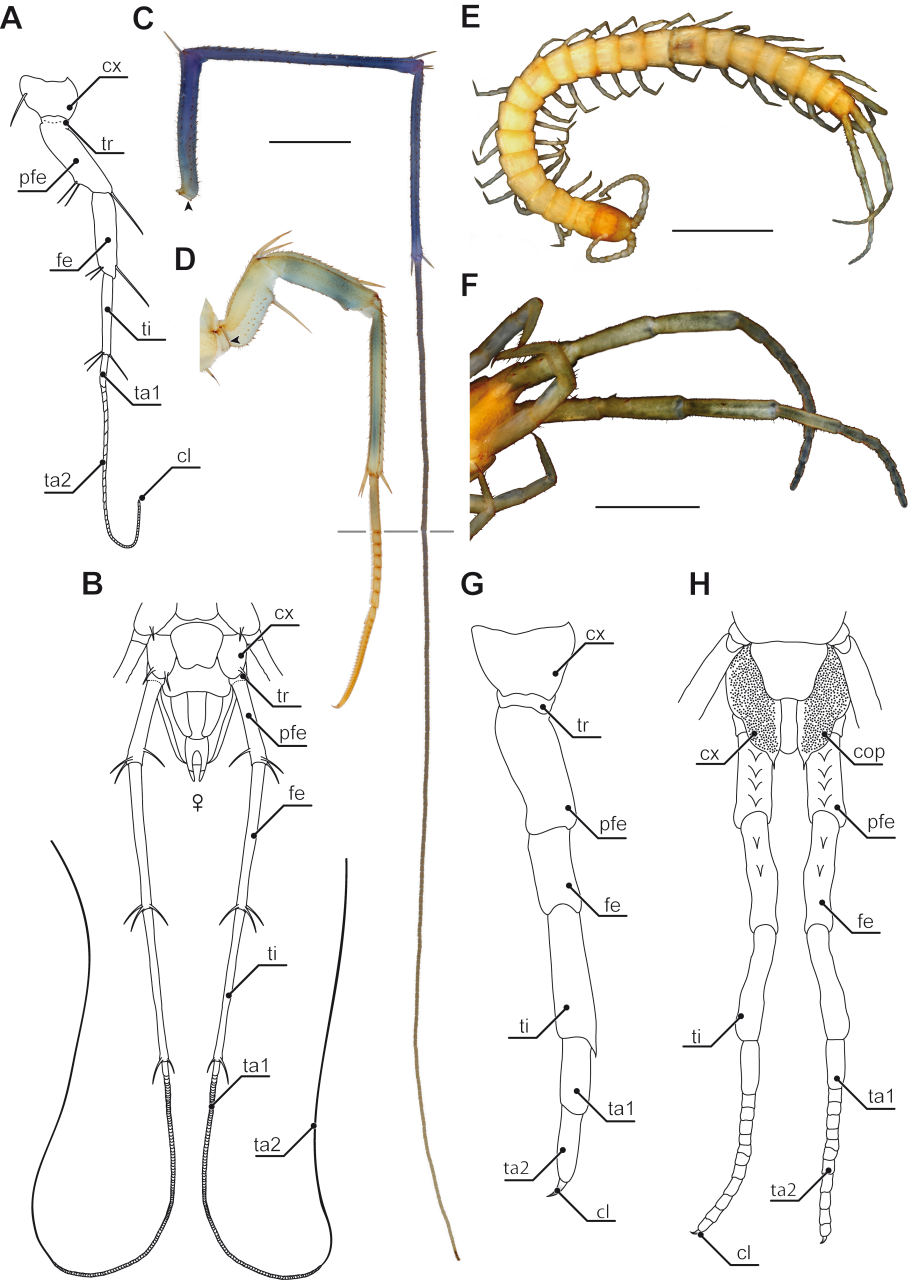
Ultimate legs in Scutigeromorpha and *Newportia* spp. (A) Schematic representation of walking leg 10 of *Scutigera coleoptrata*. The breaking point (for autotomization) of the leg is located between trochanter and prefemur, indicated by a dotted line (compare black arrowhead in C and D, Original). (B) Schematic representation of the posterior trunk and ultimate legs of a female *S. coleoptrata* (view from ventral, modified after Minelli & Koch, 2011). (C) Ultimate leg of *S. coleoptrata* (compare B, Original). Tip of tarsus 2 is incompletely regenerated. Transition of tarsus 1 and 2 indicated by grey line. (D) Walking leg 10 of *S. coleoptrata* equally scaled to the ultimate leg** (compare A, Original). Transition of tarsus 1 and 2 indicated by grey line. (E) Habitus of *Newportia monticola* Pocock, 1890 (view from dorsal, Original). Note the comparable length of antennae and ultimate legs. (F) Close up of ultimate legs with multi-annulated tarsus of *N. monticola* (view from ventral, compare H, Original). (G) Schematic representation of walking leg 10 of *Newportia longitarsis* (Newport, 1845) and (H) Schematic representation of the posterior trunk and ultimate legs of *N. longitarsis* (view from ventral, compiled after Schileyko & Minelli, 1998). Scale bars: A, C 500 µm, E 250 µm, F 100 µm. Abbreviations: cl, pretarsal claw; cx, coxa; fe, femur; pfe, prefemur; ta1, tarsus 1; ta2, tarsus 2; ti, tibia; tr, trochanter.

Evolutionary transformations of myriapod legs remained poorly studied for many decades. One reason for this lack of information and attention paid by researchers probably is that, as for comparison with hexapods, crustaceans, and also arachnids, myriapods with about 16,000 species described represent only a small fraction of arthropod species diversity. They are chronically understudied (Müller & Rosenberg, 2009; Rosenberg & Müller, 2009; Sombke & Edgecombe, 2014) and poorly known with respect to many organ systems. As pointed out by Sombke & Edgecombe (2014), myriapodology is a small field that lacks specialist journals and has few taxonomic and anatomical specialists. For contributions in phylogenetics, but also in general evolutionary debates however, data on myriapods are urgently needed as pivotal questions on arthropod evolution fundamentally depend on the status and systematic position of this taxon (Edgecombe & Giribet, 2007). Myriapods are exclusively terrestrial arthropods with centipedes present in the fossil record since the Upper Silurian (ca. 420 Ma) (Shear, Jeram & Selden, 1998; Edgecombe, 2011a; Haug et al., 2014). Myriapoda comprises the four taxa Chilopoda, Symphyla, Pauropoda, and Diplopoda. Amongst Chilopoda, five major lineages can be regarded as established. Scutigeromorpha (Figs. 1A; 2) are considered to be the most basal taxon and sistergroup to Pleurostigmophora. The latter comprises Lithobiomorpha and Phylactometria (Craterostigmomorpha and Epimorpha) with Epimorpha being composed of Scolopendromorpha and Geophilomorpha (compare Edgecombe, 2011b; Fernández, Edgecombe & Giribet, 2016). Contrary to the trivial name *centipedes*, literally suggesting the presence of 100 legs, adult representatives of scutigeromorph, lithobiomorph, and craterostigmomorph species possess 15 post-forcipular pairs of legs (Fig. 1A). Scolopendromorph species possess 21 or 23 pairs of legs (Fig. 3E; but also 39 or 43 pairs have been recorded in *Scolopendropsis duplicata* Chagas-Júnior, Edgecombe & Minelli, 2008), while Geophilomorpha may possess up to 191 pairs of legs, with males usually having fewer legs (Lewis, 1981; Rosenberg, 2009). Irrespective of their always odd number, centipede legs are consistently composed of six or seven podomeres (coxa, trochanter, prefemur, femur, tibia, tarsus 1 and, if present, tarsus 2) and a pretarsal claw (Fig. 1B). However, the actual podomere configuration and terminology differs between taxa, and occasionally even within different legs of single species.

Several compendia and books already addressed aspects on general biology and evolution in centipedes (Latzel, 1880; Verhoeff, 1902; Attems, 1930; Lawrence, 1953; Dobroruka, 1961; Cloudsley-Thompson, 1968; Lewis, 1981; Rosenberg, 2009; Minelli, 2011; McMonigle, 2014). The aspect of leg modification however, was mostly focused on the forcipules alone (Fig. 1C). These former first walking legs are a hallmark of centipedes. They are prominently transformed, carrying venom glands, as well as sensilla, and are used for biting, killing, as well as manipulating prey items prior to feeding (Rosenberg, 2009; Dugon, Black & Arthur, 2012), but also play an important role in grooming (Rosenberg, Brenner & Greven, 2004; Rosenberg, Brenner & Greven, 2005). Accordingly, they have been the subject of several investigations of varying scope: anatomy of the glandular system (Karlinski, 1883; Undheim & King, 2011; Dugon & Arthur, 2012), sensilla (Ernst & Rosenberg, 2003), morphology of the forcipular appendage (Dugon, Black & Arthur, 2012; Haug et al., 2014; Maruzzo & Bonato, 2014), and toxins (Undheim et al., 2014; Undheim, Fry & King, 2015). In comparison with the remaining myriapod taxa it is clearly evident that the forcipules were derived from a walking leg and became further transformed along the centipedes’ stem lineage (Edgecombe, 2011b; Haug et al., 2014). Many podomeres being more or less distinctly distinguished on forcipules are fusion products of podomeres of the typical walking leg (Figs. 1B and 1C). The morphology of single forcipular elements, however, varies considerably in different centipede taxa. Haug et al. (2014) drew an evolutionary scenario proposing transformations in major centipede lineages. In Scutigeromorpha, the forcipule still appears leg-like. In Pleurostigmophora (all remaining centipedes) however, the proximal elements (coxosternite) are medially coalesced and in Epimorpha (Scolopendromorpha and Geophilomorpha) the distal podomeres (trochanteroprefemur, femur, tibia, and tarsungulum) possess shared joints (Fig. 1C) (Edgecombe, 2011b; Dugon, Black & Arthur, 2012; Haug et al., 2014).

In addition to the forcipules, and despite its taxonomic significance, it is the posterior end of the centipedes’ body that shows a considerable structural disparity and functional diversity of appendages. Apart from the gonopods, it is the last pair of legs (i.e., terminal, ultimate or anal legs; e.g., Bonato et al., 2010) that is particularly unique as no other leg in centipedes is of a comparable functional, morphological, and behavioral heterogeneity. This review sets out to summarize the current state of knowledge on ultimate legs of centipedes in terms of morphology, variability, posture, and behavioral adaptations. Along these lines, ultimate legs in centipedes may provide a promising opportunity to explore pathways of leg evolution at the interface of phylogenetic, functional, and constructional constraints.

## Methods

### Survey methodology

Literature searching aimed at collecting any published data on morphology and function on centipede ultimate legs. This search strategy was used in several databases, including PubMed, Google Scholar, YouTube, the myriapod literature database Myrialit (http://myriapodology.org/myrlit/), as well as the private database of Dr. Jörg Rosenberg. No language restrictions were applied.

### Imaging

For SEM images, specimens of *Lithobius forficatus* and *Cryptops hortensis* were collected in Greifswald (Germany), processed after the protocol by Sombke et al. (2011), and examined with a Zeiss EVO LS10 at the Imaging center of the University of Greifswald. For macro-images, specimen were fixed in 70% ethanol and analyzed using the BK PLUS Lab system (Dun Inc., http://www.duninc.com/bk-plus-lab-system.html) with a customized Canon MPE 65 mm 1–5× micro-photography lens mounted on a Canon 6D camera. Image stacks were captured with Adobe Lightroom and processed using Zerene Stacker under PMax value. Images and illustrations were produced and processed in Adobe Photoshop and Illustrator CS4.

## Morphology and Modifications of ultimate legs

Ultimate legs are special. In most adult centipede species they are the largest legs and easily noticeable by their shape, and by the way they are hold and moved in relation to regular walking legs (Jangi, 1964) (Figs. 1A, 1E and 2B). With regard to their postembryonic phase of life, centipedes exhibit two distinct patterns of leg development. Representatives of Epimorpha (i.e., Scolopendromorpha and Geophilomorpha) hatch with a full complement of legs. Hence, the prospective ultimate legs are developed, but not fully shaped until the adult stage. However, exceptions have been shown in Geophilomorpha suggestinga postembryonic addition of segments and legs (compare Verhoeff, 1902; Misioch, 1978; Minelli & Sombke, 2011; Brena & Akam, 2012; Brena, 2014). In contrast, representatives of Anamorpha (i.e., Scutigeromorpha, Lithobiomorpha, and Craterostigmomorpha) hatch with less than the adult number of legs (e.g., in *Scutigera coleoptrata* (Linnaeus, 1758), the first post-embryonic stage possesses 4 pairs of legs) and the number gradually increases during early molts (Lewis, 1981), with each last pair of legs still participating in locomotion. We therefore will only consider the last pair of legs of adults as fully developed ultimate legs.

In most species, ultimate legs are composed of those seven podomeres listed above (Figs. 1B and 1D). However, in some taxa (e.g., *Scolopendra* sp.) they might be composed of fewer or more podomeres (Verhoeff, 1902). Also, the pretarsal claw may be reduced in ultimate legs of Scutigeromorpha, as well as in some Scolopendromorpha and Geophilomorpha (Verhoeff, 1902; Lawrence, 1953; Edgecombe & Giribet, 2006). The morphology of ultimate legs and its podomeres thus often holds a high taxonomical value (Verhoeff, 1902; Attems, 1930; Shelley, 1990; Shelley, 2002; Shelley & Mercurio, 2005; Schileyko, 2009; Schileyko, 2013; Chagas-Júnior, 2011; Chagas-Júnior, 2012; Chagas-Júnior & Bichuette, 2015; Martínez-Muñoz, Dolejš & Kronmüller, 2016; Siriwut et al., 2016). Examples are species of the genus *Theatops* which possess different patterns of spurs on the prefemora and femora (Shelley, 1990), or species of the genus *Newportia* displaying a specific annulation of tarsi (Schileyko, 2013). As the importance of species-specific characters of ultimate legs has been covered in detail in a series of taxonomic studies, it is therefore beyond the scope of this review.

The ultimate legs of centipedes are never or rarely used for locomotion. Due to the morphology of the coxa, they are always held more or less in parallel to the body’s longitudinal axis (e.g., Figs. 1A and 3B; Jangi, 1961; Kaestner, 1963; Lewis, 1981; Kronmüller, 2013). While in walking legs the coxopodite is still recognizable in the pleura in form of various coxal sclerites, coxal and pleural components are completely fused to a coxopleura in ultimate legs (Fig. 1E). Also, the Y-shaped sclerotization between coxa and telopodite of the walking leg is absent in ultimate legs (Manton, 1958a; Manton, 1958b). As the flexible interpodomeric cuticular membranes are most extensive ventrally and become gradually shorter dorsally, all interpodomeric movements of the telopodite are mostly restricted to elevation and depression (Fig. 1E) (Manton, 1958a; Manton, 1958b; Manton, 1977). A series of hinge joints, whose muscles cause flexures only, is positioned dorsally along the rest of the leg (Fig. 1E). While the centipede is running, the ultimate legs are kept lifted up and often outward (Figs. 1E and 1F). Jangi (1961) gave a detailed description of the ultimate leg morphology and anatomy in *Scolopendra morsitans* Linnaeus, 1758 showing that innervation and muscular equipment equally render them rather unfit for a locomotory function. As pointed out by Jangi (1961) and Manton (1977) for walking legs, the number of extrinsic muscles is correlated with a species’ movement speed. For instance, Scolopendromorpha possess 18 muscles while the fast-running Scutigeromorpha possess 34 (Manton, 1965; Manton, 1977; Manton, 1979). In contrast, ultimate legs in *S. morsitans* are equipped with merely seven extrinsic muscles (Jangi, 1961).

**Figure 4:**
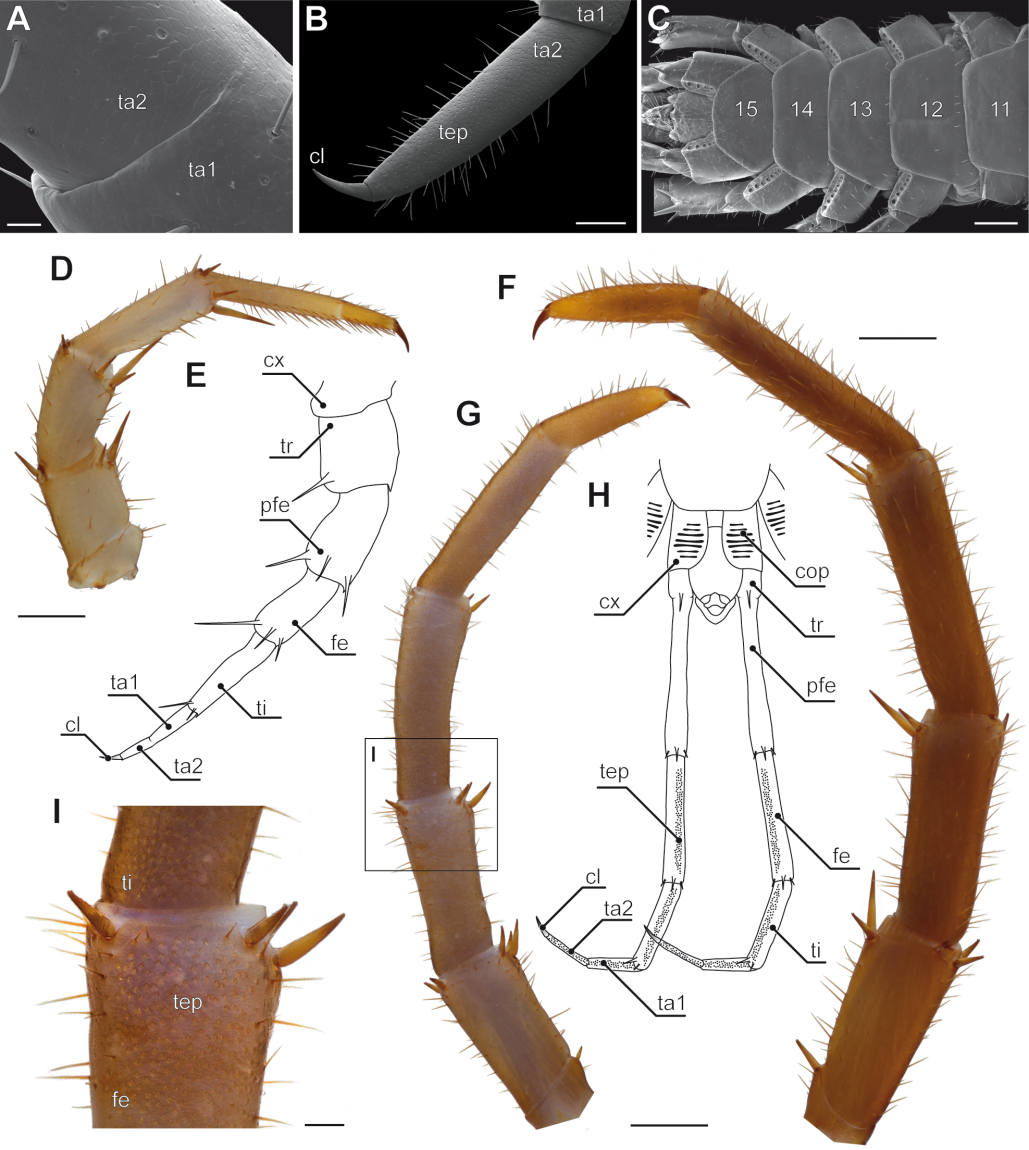
Aspects of ultimate legs in *Lithobius forficatus*. (A) Incomplete separation of tarsus 1 and 2 of the walking leg 10 (SEM, Original). (B) Tarsus 2 and pretarsal claw of the ultimate leg, medial side with pores of telopodite glands (SEM, Original). (C) Posterior trunk from ventral. Coxae of legs 12 to 15 (ultimate leg) each possess a row of coxal pores (SEM, Original). (D) Walking leg 10. Note the incomplete separation of tarsus 1 and 2 indicated by the whitish interpodomeric cuticular membrane (Original). (E) Schematic representation of walking leg 10 (Original). (F) Left ultimate leg from lateral (Original). (G) Right ultimate leg from medial (Original). Note the milky appearance of the cuticular surface due to the pores of telopodite glands. (H) Schematic representation of the posterior trunk with ultimate legs (view from ventral, modified after Minelli & Koch, 2011). (I) Inset as indicated in G showing the pores of telopodite glands on femur and tibia. Scale bars: A 20 µm, B 200 µm, C, D, G, F 500 µm, I 100 µm. Abbreviations: cl, pretarsal claw; cop, coxal pores; cx, coxa; fe, femur; pfe, prefemur; ta1, tarsus 1; ta2, tarsus 2; tep, pore of telopodite glands; ti, tibia; tr, trochanter, 11–15 sternites of leg-bearing segments 11 to 15.

Accordingly, the last pair of legs always holds a literally outstanding special status in terms of posture and morphology when compared to ‘ordinary’ walking legs. Hence, we present the external morphology of the 10th walking leg and ultimate leg in Geophilomorpha (Figs. 1B vs. 1D), Scutigeromorpha (Figs. 3A vs. 3B), Lithobiomorpha (Figs. 4E vs. 4H), and various Scolopendromorpha (Figs. 3G vs. 3H; 5B vs. 5C and 5F vs. 5H; 6B vs. 6C). As a general rule, several not mutually exclusive themes of morphological modifications and behavioral adaptations can be distinguished. Many species possess elongated ultimate legs, some species possess pincer-like ultimate legs, and in many species sexual dimorphisms do occur. Ultimate legs may have a raptorial or defensive function. In addition, glandular pores are much more often present on ultimate legs than on walking legs (Verhoeff, 1902), and an involvement in courtship behavior was described in some species.

Tracing evolutionary stages of ultimate leg morphology in centipedes is difficult as respective data on fossil centipedes is lacking, and the number of described fossil specimens is very low (Edgecombe, 2011a). Concerning Scutigeromorpha (e.g., the carboniferous *Latzelia primordalis* Scudder, 1890), we have little to no information on ultimate legs as these fragile legs are either detached or not preserved during the taphonomic process (compare Shear, Jeram & Selden, 1998; Edgecombe, 2011a; Haug et al., 2014). A few well preserved scolopendromorph representatives from the Carboniferous (e.g., *Mazoscolopendra richardsoni* Mundel, 1979) however, indicate that these animals already possessed enlarged ultimate legs (compare Haug et al., 2014). Also, Baltic amber fossils show a strong correspondence in ultimate leg morphology to extant representatives (Koch & Berendt, 1854; Gröhn, 2015).

**Figure 5:**
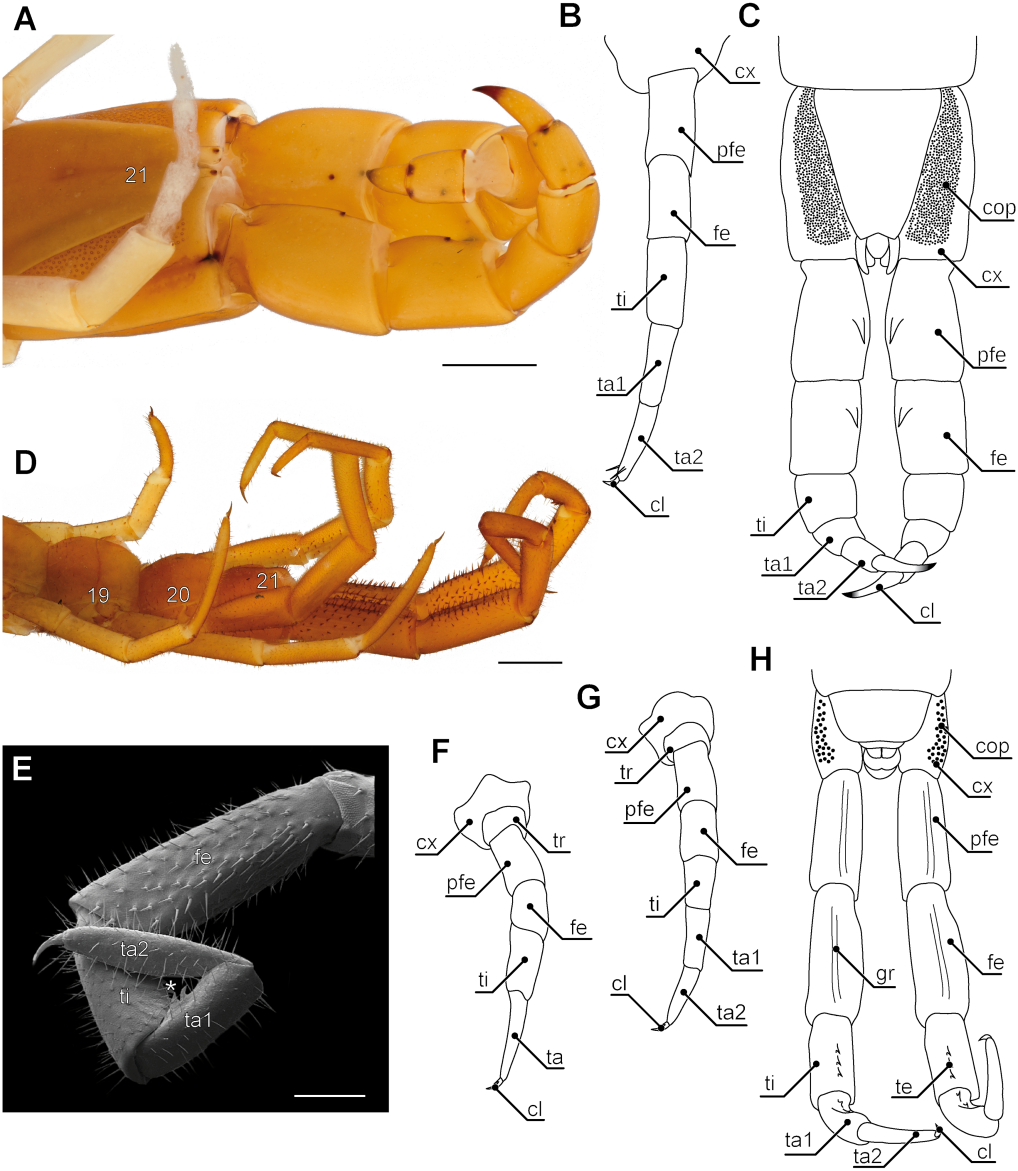
Aspects of ultimate legs in *Theatops* spp. and *Cryptops hortensis*. (A) Ultimate legs of *Theatops erythrocephalus* C.L. Koch, 1847 (view from ventrolateral, Original). Tarsal podomeres are slightly detached. The ventral coxopleura is covered with coxal pores. (B) Schematic representation of walking leg 10 in *T. erythrocephalus* (Original), and (C) the posterior trunk with ultimate legs in *Theatops californiensis* Chamberlin, 1902 (modified after Shelley, 1997). (D) Posterior trunk and walking legs 19, 20, as well as ultimate legs of *Cryptops hortensis* (view from ventrolateral, Original). (E) Left ultimate leg “claw” of *C. hortensis* (view from medial, SEM, Original). Note the tibial and tarsal ‘sawteeth’ (asterisk). Please note the conflicting terminology of podomeres and compare also Fig. 6A. (F–G) Schematic representation of walking leg 19 (F) and walking leg 20 (G). Note the division of the tarsus in walking leg 20 (Originals). (H) Schematic representation of the posterior trunk and ultimate legs (view from ventral, Original). Note the ventral grooves on prefemora and femora, as well as the ‘teeth’ on tibia and tarsus 1. Scale bars: A 1 mm, D 500 µm, E 250 µm.Abbreviations: cl, pretarsal claw; cop, coxal pores; cx, coxa; fe, femur; gr, grooves on prefemora and femora; pfe, prefemur; ta, tarsus; ta1, tarsus 1; ta2, tarsus 2; te, ‘teeth’ of tibia and tarsus 1; ti, tibia; tr, trochanter; 19–21 sternites of leg-bearing segments 19 to 21.

**Figure 6:**
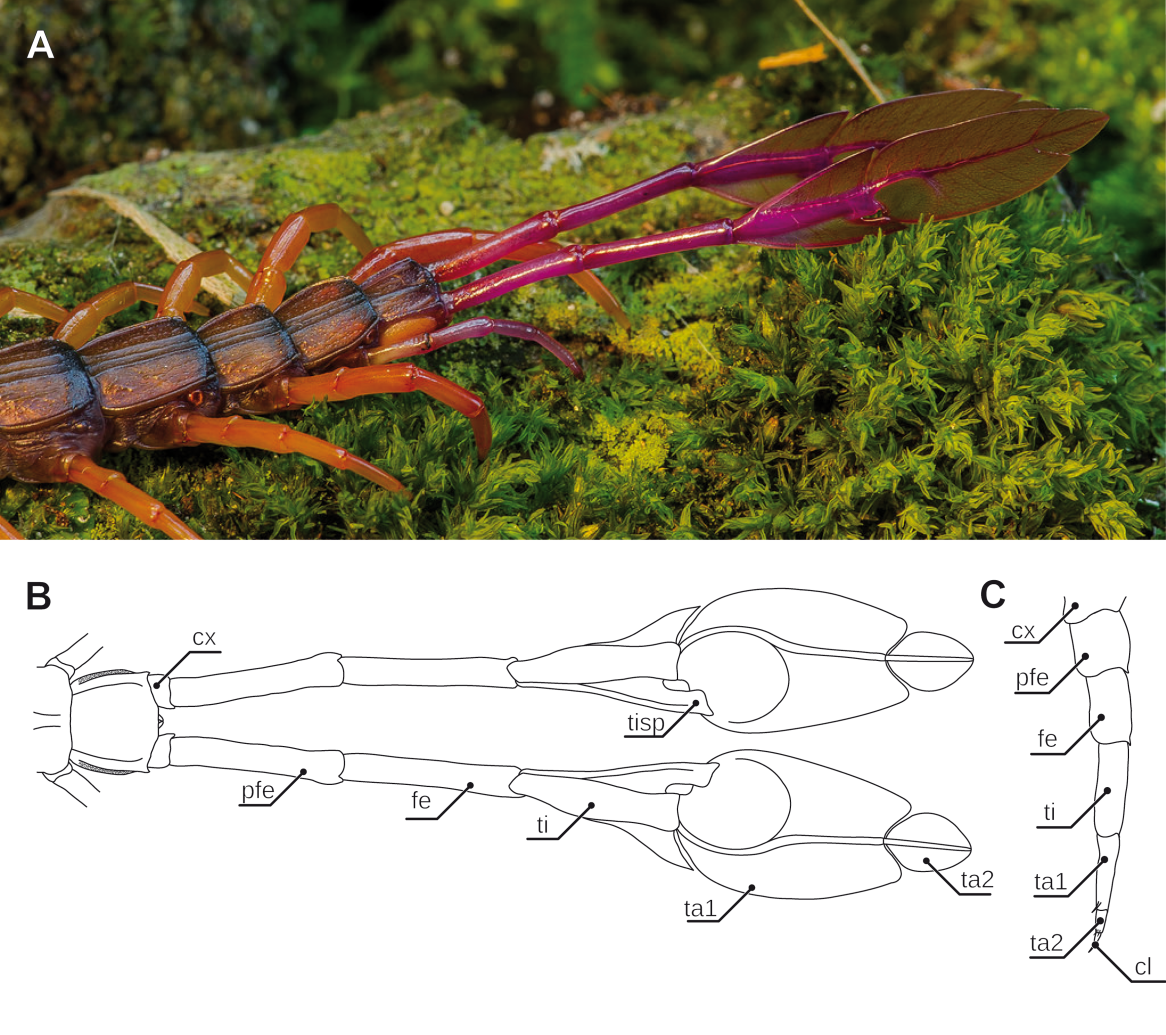
Aspects of ultimate legs in *Alipes* spp. (A) Posterior trunk with leaf-like ultimate legs in *Alipes multicostis* Immhoff, 1854 (Original A. Ruppert). (B) Schematic representation of posterior trunk and ultimate legs in *Alipes* spp. (compiled after *Alipes grandidieri*; (Iorio, 2003) and *Alipes crotalus* (Gerstaecker, 1854); and own data). Note that the distal podomeres are turned with medial sides facing upward in order to illustrate the leaf-like appearance. (C) Schematic representation of walking leg 10 in *A. crotalus* (Original). Abbreviations: cl, pretarsal claw; cx, coxa; fe, femur; pfe, prefemur; ta1, tarsus 1; ta2, tarsus 2; ti, tibia; tisp, tibial spur.

### Ultimate legs in motion and balance

As pointed out above, ultimate legs of centipedes in most cases do not participate in locomotion in terms of propulsion. However, they may still play a role in the process by stabilizing the body while running. Concerning Lithobiomorpha, Verhoeff (1902) described that they are always stretched backwards symmetrically, which can also be observed in other fast-running centipedes. Most centipedes are agile and elegant runners that are able to perform fast turns. Thus, when a centipede turns to one side, the ultimate legs move to the other side which by conservation of angular momentum results in a faster turn. This is corroborated by experiments with one removed ultimate leg: the remaining ultimate leg is kept in the median of the body in order to keep the center of mass and hence its balance (Verhoeff, 1902). Verhoeff also pointed out that the faster a centipede species is able to run, the longer the ultimate legs are.

### Annulation of ultimate leg tarsi

Especially in Scutigeromorpha (Verhoeff, 1902; Edgecombe & Barrow, 2007; Edgecombe, 2011c), but also in some Lithobiomorpha (e.g., *Cermatobius*; Ma, Song & Zhu, 2007) and Scolopendromorpha (especially *Newportia*; e.g., Schileyko & Minelli, 1998), the ultimate legs are significantly elongated, achieved by a secondary fragmentation of the tarsal podomeres (Figs. 1A and 3). A considerable and along the body axis consecutively increasing degree of annulation is encountered on the tarsi of the walking legs of Scutigeromorpha, which is considered an adaptation to fast running (Manton, 1977). However, the elongation of ultimate legs exceeds them by far (Figs. 2B; 3A–3D). Usually tarsus 2, but also tarsus 1 (in Scutigeromorpha), is subdivided into a multitude of annuli, literally transforming the leg into an antenniform appendage that bears an as yet unspecified array of sensilla and trichomes. In various species of Scutigeromorpha, the ultimate legs are about twice as long as the walking legs and may reach or even surpass the length of their antennae (Snodgrass, 1952; Lewis, 1981; Sombke et al., 2011) (Fig. 1A). Also, the differentiation of the two tarsi in up to 500 annuli (i.e., tarsomeres; Figs. 3B, 3C) in *Scutigera coleoptrata* is well within the range of the number of antennomeres (Latzel, 1880; Verhoeff, 1902; Sombke et al., 2011; note that Verhoeff only counted 200). In other species like *Pilbarascutigera incola* (Verhoeff, 1925) 359 tarsomeres have been counted (Edgecombe & Barrow, 2007) while in *Ballonema gracilipes* Verhoeff, 1904 the tarsi are composed of 144 annuli (Verhoeff, 1902). Several representatives of Scolopendromorpha also possess elongated ultimate legs as a result of the development of tarsus 2 annuli of varying proportions (up to 39 annuli; Figs. 3E, 3F and 3H). While in many species of the genus *Newportia* the ultimate legs are about as long as their antennae (Fig. 3E), they are at least twice as long in *Newportia stoevi* (Schileyko, 2013) and in *Tidops* species (Edgecombe & Bonato, 2011; Chagas-Júnior, 2011; Schileyko, 2013).

An increase in podomere number and/or an elongation of arthropodia achieved by intercalary annulation is a widespread phenomenon across Arthropoda (e.g., Amblypygi; Weygoldt, 1996 or Caridea; Boxshall, 2004). Annulation mostly applies to sensory appendages, for instance the antennae (Fig. 2A; McLaughlin, 1982; Boxshall, 2013). By adding components, the potential array and range of sensory organs, and hence its sensor span, as well as maneuverability can easily be increased. According to Verhoeff (1902), Kaestner (1963), and Kronmüller (2013), the multi-annulated ultimate legs of Scutigeromorpha and some Scolopendromorpha are rarely used for locomotion, but still might be involved in other ways as mentioned above. In fact, in scutigeromorph species the legs are most likely used as a multifunctional or exclusively sensory appendage. Already Verhoeff (1902), Verhoeff (1935) and Kaestner (1963) speculated about a possible sensory function as feeler or posterior antenna, although only sparse and anecdotal information about putative sensory structures are available. Our own observations on *Scutigera coleoptrata*, however, seem to confirm this assumption. The ultimate legs are always held in parallel or oblique to the substrate, similar to the posture of the antennae (Fig. 2; Sombke et al., 2011), solely touching the ground in a “probing” manner. Occasionally, the animals can be observed making fast twitching up and downward movements with their ultimate legs which, at least in its appearance, bears a strong resemblance to the antennal flicking of crustaceans (e.g., Koehl, 2011; Waldrop & Koehl, 2016, and own observations). This behavior can be observed in specimens that just captured prey, took a resting position, or got excited by any kinds of disturbance. However, the same holds true for various lithobiids lifting their ultimate legs while running or being disturbed (Verhoeff, 1902).

### Variations on a theme—elimination, addition, and transformation

In many centipedes, ultimate legs are distinctly longer than walking legs and are more or less, sometimes quite substantially, thickened. Likewise, conspicuous modifications of podomere structure are frequent. Often they are covered with a multitude of sturdy spurs, spines, and trichomes (Di et al., 2010; Edgecombe & Bonato, 2011), but also with a higher number of putative sensilla (Lewis, 2010), as compared to ordinary walking legs. In some species, podomeres are fused forming a compound podomere (i.e., trochanteroprefemur in e.g., *Lithobius* sp. or *Scolopendra* sp.; Lewis, 1981; Kronmüller, 2013). In *Lithobius forficatus* (Linnaeus, 1758), the tarsi of walking legs feature an incomplete fusion while they are clearly distinct in ultimate legs (Figs. 4A, 4B, 4E, 4H). In all Scolopendromorpha, the trochanter of the ultimate leg is more or less vestigial (Verhoeff, 1902). In *Scolopendra morsitans*, the two small trochanteral sclerites are not visible externally, and the smaller ones can only be deflected outwards which leads to a spreading of the ultimate legs (Fig. 1F).

**Figure 7:**
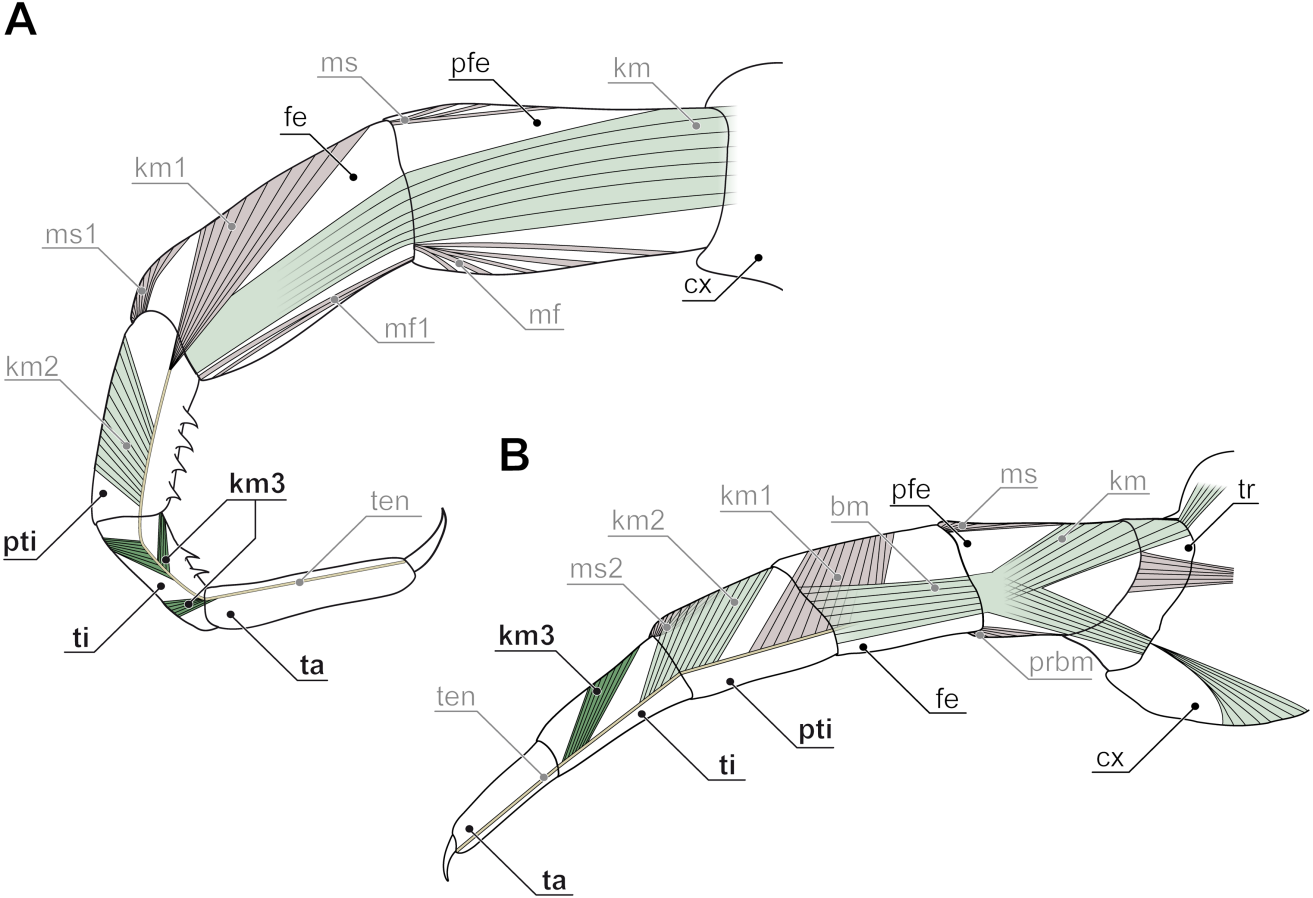
Intrinsic muscles in centipede ultimate legs (modified after Verhoeff, 1903). (A) Ultimate leg of *Cryptops hortensis* (Scolopendromorpha). According to Verhoeff (1903), only the tarsus is devoid of intrinsic musculature. Consequently, Verhoeff proposed the podomere ‘pretibia’ between femur and tibia. (B) Ultimate leg of *Geophilus carpophagus* (Geophilomorpha). In this species, Verhoeff (1903) also detected intrinsic muscles in the penultimate podomere. Consequently, he proposed a ‘pretibia’ between femur and tibia. For details see discussion on modifications of ultimate leg podomeres. Abbreviations: bm, indirect muscle (*Brückenmuskel*); cx, coxa; fe, femur; km, muscles associated with the tendon (*Krallenmuskeln*); km3, tendon muscle of tibia; kmt, tendon muscle of trochanter; mf, and ms, dorsal and ventral direct muscles; pfe, prefemur; prbm, indirect muscle of prefemur and trochanter; pti, pretibia; ta, tarsus; ten, tendon; ti, tibia; tr, trochanter.

Ultimate legs might also be further segmented by the addition or fragmentation of podomeres. An exception of the general podomere configuration is present in cryptopid Scolopendromorpha. Whereas walking legs 1–19 of *Cryptops hortensis* (Donovan, 1810) possess a single clawed tarsus (Fig. 5F), leg 20 and perhaps also leg 21 possess two tarsal elements (Figs. 5D, 5E, 5G, 5H). The reason for the uncertainty concerning leg 21 came to known as the pretibia-hypothesis (sensu Verhoeff, 1903). According to Verhoeff’s argumentation, the penultimate podomere of ultimate legs actually represents a true segment due to the presence of intrinsic muscles (Fig. 7A). Thus, the podomere that has been hitherto identified as tarsus 1 (compare Fig. 5H) actually is the tibia, and what has been assumed to be the tibia, consequently has to be interpreted as a pretibia (or postfemur, Fig. 7A). This is in contrast to ultimate legs in *Scolopendra morsitans*, where the two true tarsi (i.e., devoid of intrinsic muscles) are present (Jangi, 1961). A fragmentation of the ultimate leg tibia into two podomeres was, however, also proposed by Verhoeff (1903) for the geophilomorphs *Mecistocephalus* sp. and *Geophilus carpophagus* Leach, 1815 (Fig. 7B). Despite the age of Verhoeff’s publication, his hypothesis and related terminology never received any greater deal of attention. In fact, most of the literature holds on to the conventional terminology of two tarsal podomeres (Brölemann, 1930; Lewis, 2010; Edgecombe & Bonato, 2011; Kronmüller & Lewis, 2015). Moreover, ultimate legs of cryptopids feature another structural peculiarity: prominently toothed ridges on the ‘tibia’ and ‘tarsus 1’, and the ability to move both against each other in a penknife-like manner renders the hypothesis of a raptorial leg a reasonable assumption (see Figs. 5D, 5E, 5H; 7A and below), although this idea has been challenged by several authors (Lawrence, 1953; Lewis, 2010; Kronmüller & Lewis, 2015).

Enlarged and remarkably thickened ultimate legs with long pretarsal claws are found in species of the genera *Theatops* and *Plutonium* (Figs. 5A and 5C) (Shelley, 1997). Apart from short notes by Verhoeff (1902) and Manton (1965), there is only one report on what function the ultimate legs of these species (living deep in rock fissures) might serve. While Schileyko (2009) assumed that these legs act as pincers, Lewis (2010) suggested they rather form powerful hooks effective in attacking prey or repelling opponents, but are of no use as pincers. However, a video posted on YouTube (UnicoCelula, 2012) clearly corroborates Schileyko’s assumption. Further examples are representatives of the rare Ectonocryptopinae (i.e., *Ectonocryptoides sandrops* Schileyko, 2009, *E. quadrimeropus* Shelley & Mercurio, 2005, and *Ectonocryptops kraepelini* Crabill, 1977) (Shelley & Mercurio, 2005; Shelley & Mercurio, 2008; Schileyko, 2009). Irrespective of their uncertain classification as possible subgenera of *Newportia* (Vahtera, Edgecombe & Giribet, 2013), the ultimate legs consist of only four or five podomeres, with tarsus 2, as well as pretarsal claw being absent or present, depending on the species. However, all share a distal-most podomere prominently appearing bulbous or club-shaped. The specific biological function of these legs is a matter of pure conjecture. As species descriptions did not account for the sex of holotypes, a possible sexual dimorphic relevance as commonly seen in Geophilomorpha (see below) cannot be ruled out.

The most peculiar example of podomere transformation is found in *Alipes* spp.: while juveniles possess “rather normal” ultimate legs (cf. Verhoeff, 1902), in adult specimen the usually claw-less ultimate legs are colorful (aposematism), distinctly elongated, and tibia, as well as tarsus 1 and 2 are flamboyantly broadened dorsoventrally, reminiscent of a leaf on a twig (mimesis; Figs. 6A and 6B; note that Attems (1930) identified a small pretarsal protuberance). Upon disturbance by a predator yet not by conspecifics, the animal swings the entire leg in a wide horizontal and the distal podomeres in a short vertical amplitude. Additionally, the legs emit an audible rustling noise of 10–80 kHz, not unlike a rattle snake or longicorn beetle (stridulation; Skovmand & Enghoff, 1980; Iorio, 2003; Ruppert, 2015). While Verhoeff (1902) suggested that the sound is a passive result of scraping against the leaf litter, Lawrence (1975) believed that the sound is produced by rubbing both legs against each other. However, as it already has been assumed by Gerstäcker (1854) and later been validated by Skovmand & Enghoff (1980), it is short repetitive bursts of rasping movements of a file on the tibial spur against a bulgy scraper on tarsus 1 that produces the sound. Lawrence (1953), as well as Skovmand & Enghoff (1980) hypothesized that this behavior most likely serves to deter, or if this proves ineffective, even excite the attention of a charging predator. In response to an attack, the ultimate legs are then readily autotomized and continue, or if not doing so already, immediately initiate to rustle, twitching for up to several minutes and thus allowing the animal to escape from the distracted predator (Lewis, 1981).

### Capture of prey

Some reports on scolopendromorph species indicate that they use their thickened ultimate legs as claws or pincers, holding forceps, or even as a defensive weapon (Verhoeff, 1902; Kaestner, 1963; Eason, 1964; Manton, 1965; Bücherl, 1971; Schileyko, 2009; Kronmüller, 2013; Kronmüller & Lewis, 2015). Bücherl (1971) observed *Scolopendra* sp. to capture its prey using the ultimate legs. This observation was recently corroborated by Kronmüller & Lewis (2015) showing that individuals being touched at the rear third of their body using forceps, raised and lowered their ultimate legs, and even occasionally attacked the forceps using their ultimate legs. Moreover, although the ultimate legs are not likely used for propulsion, they may serve at least as an anchorage. Several species of *Scolopendra* (e.g., *S. subspinipes* Leach, 1815, *S. abnormis* Lewis and Daszak, 1996 or *S. gigantea* Linnaeus, 1758) are able to use their ultimate legs to fasten themselves to any substrate suitable (fabric, rock, plants or a camera tripod; Carpenter & Gillingham, 1984; Kronmüller & Lewis, 2015), and swing their bodies quickly from side to side in order to seize prey with their remaining legs. The most impressive example is the bat-catching *S. gigantea*. Clinging from the ceiling of (Venezuelan) caves using its last five to eight pairs of legs, the centipede is able to subdue and devour prey that is substantially larger in mid-air (Molinari et al., 2005).

Although scutigeromorph species have not been observed to use their ultimate legs for capturing prey, it is noteworthy that the walking legs apply a very remarkable behavior in prey capture and holding: the flagelliform tarsi 2 often are twined around a prey item like a lasso. By doing so, the animal can capture and hold more than one item at a time while it is still able to move (Haake, 1885; Haake, 1886; Verhoeff, 1935; Lewis, 1981).

### Defense and secretion

Morphological characteristics of presumptive raptorial ultimate legs can also be interpreted as modifications in favor of defensive strategies. Verhoeff (1902) proposed that in Lithobiomorpha and Scolopendromorpha forcipules at the front and ultimate legs at the back may be used in attack and defense. Scolopendromorphs and lithobiomorphs elevate their ultimate legs in a defensive display—also in vigorous defense of their offspring (Heymons, 1901). If a female is disturbed, she spreads the ultimate legs and tries to dissuade the invader. This behavior is supposed to be an autonomous response as it can also be observed in decapitated specimens. The ultimate legs in *Cryptops* spp. were thought to work as holding forceps or even as raptorial legs (Figs. 5D and 5E; Verhoeff, 1902). Verhoeffs’ view however, has been challenged by Lewis (2010) who suggested a more defensive function instead. Already Lawrence (1953) noted that during an attack launched by a predator the ultimate legs of *Cryptops* spp. are readily autotomized, contradicting a role in grasping prey, but rather constitutes an efficient defensive strategy. One could argue that every morphological character once established must perform a function enabling a given animal to survive. By taking also into consideration the morphological peculiarities demonstrated by cryptopid ultimate legs (see above; Verhoeff, 1902; Lewis, 2010), we conclude that Verhoeff’s observations and interpretation cannot be dismissed as easily. A plausible explanation might thus be that a given predator is grasped any place suitable (antennae, legs, etc.) and the legs are autotomized in an attempt to escape. There are few description of detached legs that perform wriggling movements (Lewis, 1981) that can be interpreted as distraction of a given predator as it has been suggested for *Alipes* spp. (see above). In general, a breaking point can be located distally of the coxa as in Scolopendromorpha and Lithobiomorpha, or the trochanter as in Scutigeromorpha (Figs. 3A and 3B; Verhoeff, 1902; Maruzzo et al., 2005).

In Lithobiomorpha, another defensive performance is achieved by the last four pairs of legs—but is mostly achieved by the ultimate leg alone. Facing an imminent threat, lithobiomorphs perform fast up- and downward movements of mostly, but not exclusively, the ultimate legs (Verhoeff, 1902; Keil, 1975), followed by the secretion of a sticky, slowly hardening substance (Panic, 1963; Simon, 1964). The medial sides of the distal podomeres (i.e., femur, tibia, and tarsi; Figs. 4B, 4G, 4H) are associated with pores of closely aggregated telopodite glands, also termed defense glands (Rosenberg, Müller & Hilken, 2011a). Being absent in the anamorphic larval stages, they were originally interpreted as pheromone glands (Verhoeff, 1905). According to Verhoeff (1902), the femur of *Lithobius mutabilis* L. Koch, 1862 alone accommodates about 200 glandular pores, adding up to about 800 pores per ultimate leg (compare also Fig. 4B for the tarsus of *L. forficatus*). Short threads are secreted that aggregate to a single filament that can reach a length of 7 cm (Verhoeff, 1902). These glands become effective in predation avoidance as attackers, like spiders or ants, are glued by one or several of those filaments (Verhoeff, 1902; Simon, 1964; Keil, 1975). Subsequently, the centipede may then overwhelm or escape from the attacker. The fast up and down movements of ultimate legs might also induce the hurling of the secretion as observed by Verhoeff (1902) in a staged, but not entirely unlikely encounter of *L. forficatus* and a female lycosid spider. Moreover, the biological importance of telopodite glands is demonstrated by its redundancy. In case the ultimate legs are autotomized or lost otherwise, leg pairs 12, 13, and 14 are able to maintain the defensive function (Verhoeff, 1902; Lewis, 1981).

### Sensory organs

**Figure 8:**
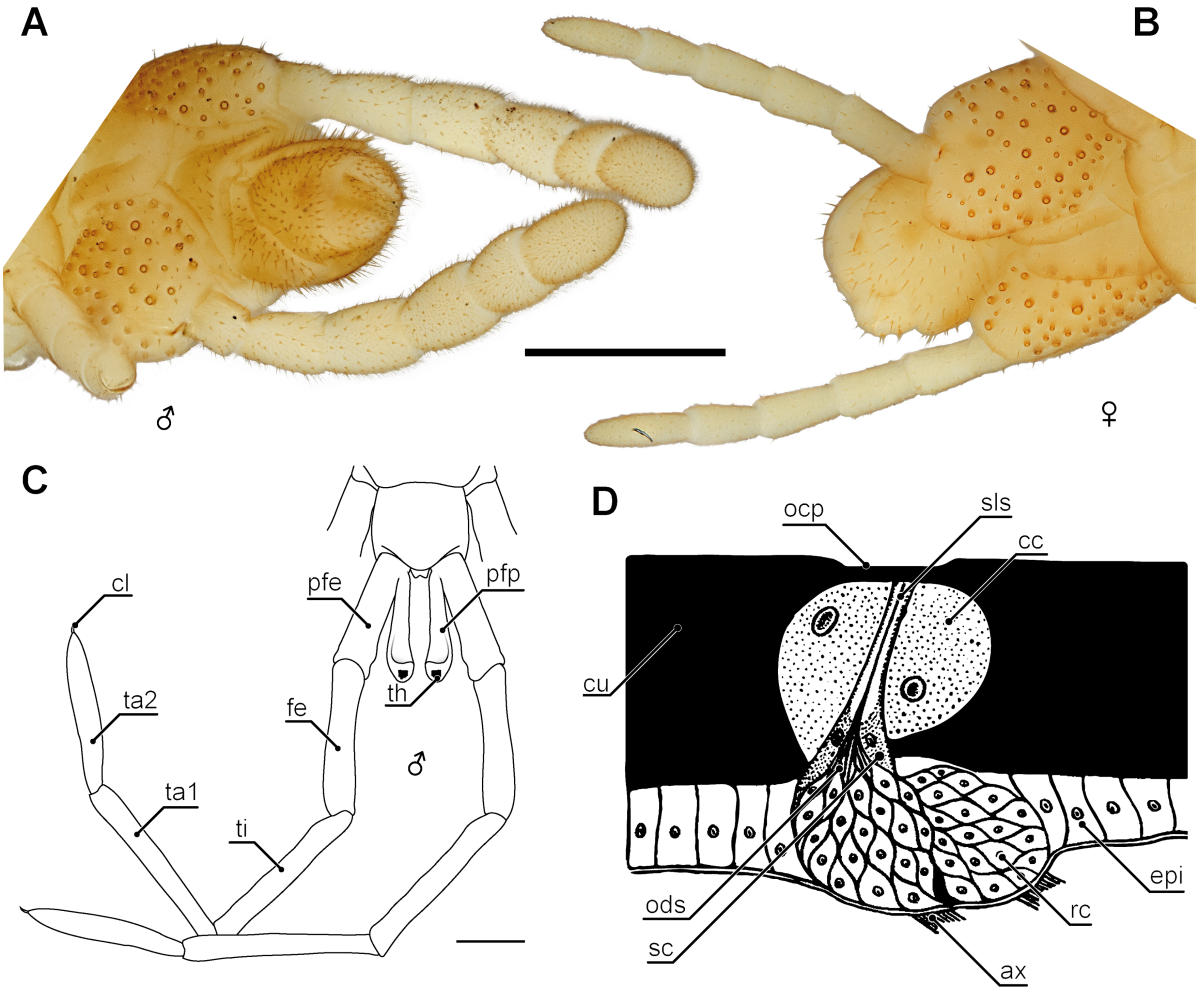
Aspects of sexual dimorphism in ultimate legs. (A) Posterior trunk and ultimate legs of a male *Haplophilus subterraneus* (view from ventral, Original). (B) Posterior trunk and ultimate legs of a female *H. subterraneus* (view from ventral, Original). Note coxal pores in both sexes. (C) Schematic representation of the posterior trunk and ultimate legs with prefemoral processes of *Otostigmus beckeri* (view from dorsal) and (D) sensory type I organ on the ultimate legs of *Himantarium samuelraji* (modified after Rajulu, 1970). Scale bars: A + B 500 µm, C 1 mm. Abbreviations: ax, axons; cc, cap cells; cl, pretarsal claw; cu, cuticle; epi, epidermis; fe, femur; ocp, outer cuticular plate; ods, outer dendritic segments; pfe, prefemur; pfp, prefemoral process; rc, receptor cells; sc, sheath cells; sls, sensillum lymph space; th, tuft of hairs; ti, tibia; ta1, tarsus 1; ta2, tarsus 2.

Albeit it has been pointed out as early as 1902 by Verhoeff that ultimate legs are “no real” legs, but rather resemble antennae, there are only a few published accounts dealing with a possible sensory function and putative sensory structures (Jangi, 1964; Rajulu, 1970; Gowri & Nageswaran, 1981). As most centipedes are capable of moving backwards, it thus was proposed that ultimate legs are modified appendages serving chemo- and mechanoreception at the posterior end of the animals’ body (Lawrence, 1953; Rajulu, 1970). The ultimate legs in Scutigeromorpha lack specific cuticular specializations usually found on walking legs such as tarsal papillae (*Tarsalzapfen*), setal cluster (*Tastborstenbüschel*der Sohle), and resilient sole-hairs (*federnde Sohlenhaare*) (Verhoeff, 1902; Verhoeff, 1935; Brölemann, 1912; Würmli, 1974; Edgecombe & Giribet, 2006). Yet, ultimate legs feature soft and steep setae (*zarte Steilborsten*) that Verhoeff (1902) homologized with a putative counterpart on the antennae. The idea that exclusively antennae-associated chemoreceptive sensilla are also present on the ultimate legs is intriguing. Inspired by the suggestion made by Lawrence (1953), Jangi (1964) demonstrated in electrophysiological experiments a mechanoreceptive function of ultimate legs of *Scolopocryptops sexspinosus* (Say, 1821). He pointed out that “it is not surprising, therefore, that the long, myriapodous, fast-running and actively hunting animal like the centipede—capable of backward locomotion—has, through structural modification, released its last pair of legs from locomotory responsibility to the reception of various stimuli in the immediate vicinity of its caudal end” (Jangi, 1964, p. 237). In addition, Rajulu (1970) reported that decapitated geophilomorphs still reacted to chemical stimuli and such reactions ceased when the ultimate legs were cut off, indicating the presence of chemoreceptors on these appendages. He investigated sensory organs on the ultimate legs of *Himantarium samuelraji* Sundara Rajulu, 1971 by histological sections (Rajulu, 1970), the results of which have been corroborated by Gowri & Nageswaran (1981) on *Haplophilus subterraneus* (Shaw, 1789). In both cases, two different sensory organs can be distinguished: Type 1 organs are exclusively present on the ventral sites of the ultimate leg tarsi and possess a thin cuticular plate (20 to 30 µm in diameter) that is slightly depressed below the level of the surrounding cuticle (Fig. 8D). Several bipolar receptor neurons innervate this cuticular plate. Their outer dendritic segments are enveloped by sheath cells that are additionally surrounded by “cap cells”. Rajulu (1970) discussed similarities with chemoreceptive sensilla placodea found on the antennae of several hexapods. Furthermore, decapitated animals were exposed to ethyl alcohol, turpentine, and ether, which resulted in flight behavior. Considering the rather irritant nature of the chemicals involved, initiation of a flight reaction is not particularly unexpected. However, when the ultimate leg tarsi were cut off or coated, no reaction to chemical stimuli was detected suggesting a chemoreceptive function of the ultimate leg tarsi and probably of the described sensory organs. In addition, Rajulu (1970) conducted electrophysiological experiments on the main nerve of isolated ultimate legs. A regular sequence of spikes was recorded when no stimulus was applied, probably due to spontaneous activity. Application of stimuli to the tarsi resulted in an increase of activity that was completely silenced when the tarsi were coated or cut off. The second type of sensory organs investigated were typical trichoid sensilla. Bipolar receptor neurons extend slightly into the shaft lumen. Similar experiments as conducted on type 1 sensory organs indicated a chemoreceptive function, whereas results of electrophysiological experiments suggested a mechanosensory function (Rajulu, 1970).

### Coxal organs

In all centipedes but Scutigeromorpha, ultimate legs feature coxal organs or coxal pores (Figs. 1D, 1E; 3H; 4C, 4H; 5A, 5C, 5H; 8A, 8B; Rosenberg, 1982; Rosenberg, 1983a; Rosenberg, 1983b; Littlewood & Blower, 1987; Littlewood, 1991a; Littlewood, 1991b; Rosenberg, Müller & Hilken, 2011b). In the majority of geophilomorphs, at least central European species, quantity and distribution of coxal pores are species-specific and thus of taxonomic importance (Rosenberg, 1982; Rosenberg, 1988a; Rosenberg, 1988b; Rosenberg, 2009). Interestingly, while in scolopendromorphs and geophilomorphs the pores are restricted to the coxae or coxopleurae of the ultimate legs, in lithobiomorphs the coxal pores are distributed on the coxae of the last four pairs of legs (i.e., 12–15; Fig. 4C; Rosenberg, 1983b; Rosenberg, 1988b; Rosenberg, 2009; Zapparoli & Edgecombe, 2011). The purpose and function of the coxal organs is not conclusively resolved and a variety of hypotheses are discussed (summarized in Rosenberg, 1983b). In fact, we only begin to understand the biological implications of these organs in *Lithobius forficatus*. While Willem (1897) assumed a role in communication and mating, Verhoeff (1902) assumed a more raptorial function. Ultrastructural investigations, however, indicated that their predominant function isthe uptake of atmospheric water via transporting epithelia (Rosenberg & Bajorat, 1984; Rosenberg, 2009) as it has been also shown for Geophilomorpha (Rosenberg, 1982). A further indication can be derived from the animals’ ecology and habitat preference as pores of coxal organs are smaller or even lacking in species living in arid environments like the geophilomorph *Mesocanthus albus* Meinert, 1870 or the scolopendromorph *Asanada sokotrana* (Lewis, 1981). However, these findings have to be regarded as open to interpretation as, based on the specific structure of the transport epithelia, lithobiomorph coxal organs were also proposed as release sites of sex-specific pheromones (Littlewood, 1983; Littlewood, 1988; Littlewood 1991a; Littlewood, 1991b; Littlewood & Blower, 1987). Altogether, previous findings and functional interpretations led to a rather ambiguous picture of coxal organ function. However, a common evolutionary origin seems likely. Based on descriptions of the anal capsule in *Craterostigmus tasmanianus* Pocock, 1902 (Craterostigmomorpha) indicating a functional uptake of water, Rosenberg, Müller & Hilken (2006) advocated for anal and coxal organs to be considered homologous across Pleurostigmophora.

### Sexual dimorphism

Sexual dimorphic characteristics of ultimate legs are found in various representatives of Lithobiomorpha, Scolopendromorpha and Geophilomorpha (Lewis, 1981; Lewis, 1985; Barber, 2009; Rosenberg, 2009). Latzel (1880) assumed a sexually dimorphic shaping of ultimate legs in Scutigeromorpha according to which males possess more tarsal annuli than females. Yet, this assumption has never been evaluated or corroborated any further, and might be the result of lesions or an incomplete regeneration. Amongst lithobiomorphs, sexually dimorphic shaping of ultimate legs is quite common, although they are only evident in mature specimens, clearly indicating a role in intraspecific communication and mate finding. Often several podomeres possess a deep groove on the dorsal side, which are lacking in females (e.g., *Lithobius dentatus* C.L. Koch, 1844). Males of *Lithobius calcaratus* C.L. Koch, 1844 and *Eupolybothrus* sp. possess conspicuous bristle tufts, spurs, and pits on various podomeres of their ultimate legs (Eason, 1973), and male *L. nodulipes* Latzel, 1880 features a noticeable longish node on tarsus 1.

Although sexual dimorphism is rather rare in Scolopendromorpha, there are few species in which the morphology of ultimate legs of males is different from females due to the occurrence of a pronounced lateral keel, a row of spines, or other cuticular protuberances (Attems, 1930; Lewis, 1981; Lewis, 2010). For example, Jangi (1961) described that male prefemora, femora, and tibiae in *Scolopendra morsitans* are dorsally flattened and possess elevated lateral and posterior margins with a small median interruption on their posterior borders. The podomeres of females have dorsally convex surfaces without such emarginations. Also in contrast to females, the sawteeth of the tibiae in various cryptopid males are arranged in multiple rows and are much more pronounced (Lewis, 2010). One highly peculiar example of sexual dimorphism in ultimate legs is found in several species of the scolopendromorph genus *Otostigmus* (Verhoeff, 1902; Chagas-Júnior, 2012). While these species lack spines of taxonomic importance, males possess a conspicuous digitiform appendix at the dorsomedial base of the prefemora, femora or tibiae, occasionally reaching about the length of the respective podomere (Fig. 8C; Chagas-Júnior, 2012; Siriwut et al., 2014). For instance, in *Otostigmus beckeri* (Chagas-Júnior, 2012) the appendix projects distally alongside the podomere, bends dorsally and ends in a knob-like top featuring a tuft of dark reddish hairs. Altogether, its appearance strongly resembles a stalked eye (Fig. 8C; Chagas-Júnior, 2012). A similar case of male-specific prefemoral processes was described in the scolopendromorph *Alipes appendiculatus* (Pocock, 1896; Edgecombe & Bonato, 2011). In Geophilomorpha, however, differences between the sexes are rather obvious. For instance in contrast to females, males of e.g., *Henia* sp. and *Haplophilus subterraneus* possess prominently thickened and hirsute ultimate legs (Lewis, 1981; Figs. 8A and 8B). Likewise, ultimate legs of adult *Strigamia* spp. males feature an intensive coverage with “hairs” (i.e., trichomes and probably sensilla) on the ventral side of several podomeres (Verhoeff, 1898; Verhoeff, 1902). Although available experimental evidence is as yet circumstantial (Klingel, 1959), ultimate legs of geophilomorphs, and certainly all centipedes, play a role in at least certain aspects of mating behavior.

### Courtship behavior and mating

**Figure 9:**
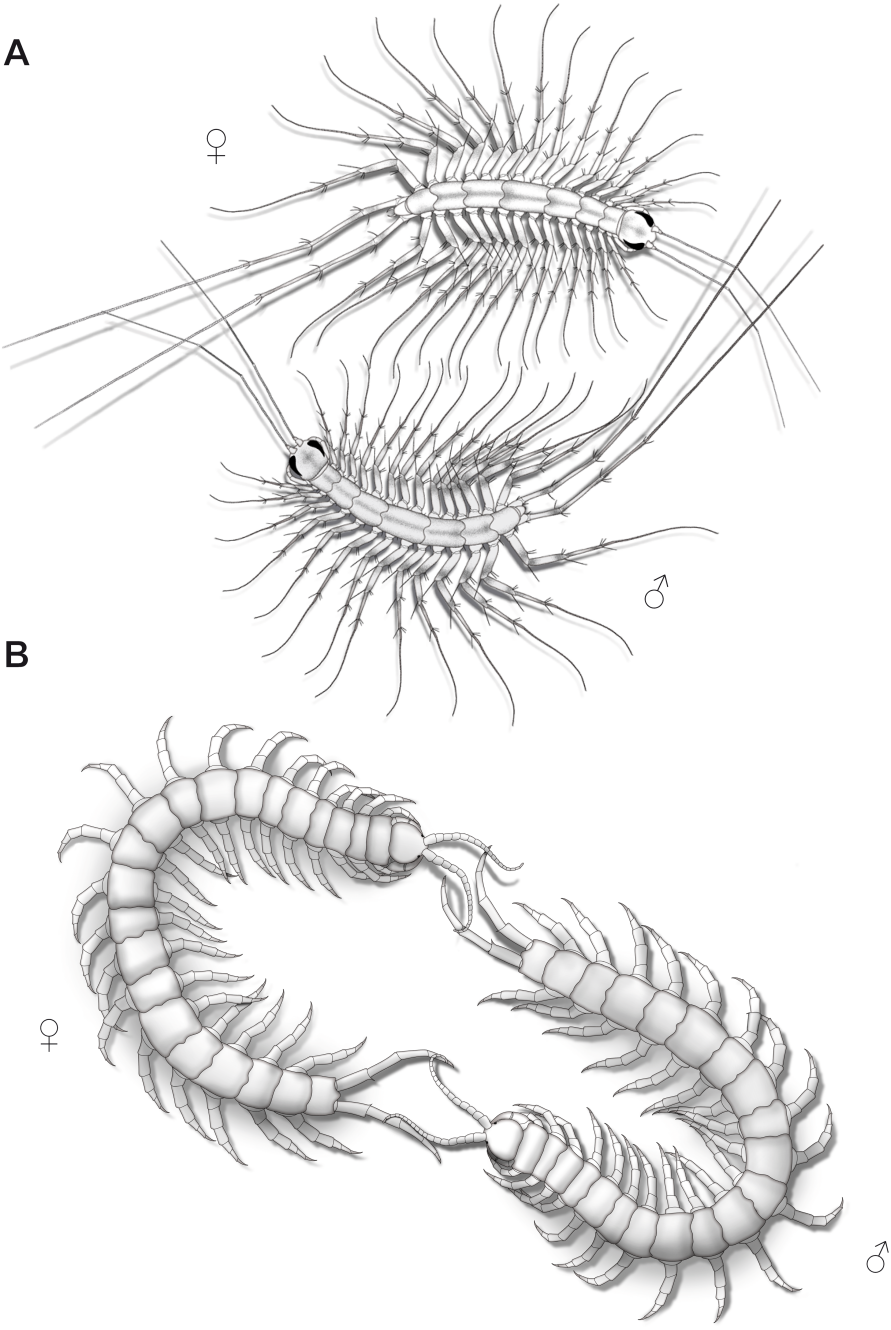
Aspects of ultimate legs during courtship behavior. (A) *Scutigera coleoptrata* (modified after Klingel, 1960a). (B) *Scolopendra cingulata* (modified after Klingel, 1960a; Radl, 1993).

In the course of mating, female and male *Scutigera coleoptrata* both slowly and repeatedly raise and lower their antennae, but also their ultimate legs, suggesting a role in intraspecific communication, conceivably pheromone sensation. Observations and illustrations by Klingel (1960a) depicting two specimens revealed a behavior that could be described as mating foreplay comprising mutual probing of the ultimate legs with the antennae, probably in order to determine the willingness of the counterpart to mate (Fig. 9A). In the scutigeromorph species *Thereuopoda decipiens* (Verhoeff, 1905), however, courtship behavior only involves the antennae and anterior-most pairs of walking legs (Klingel, 1962). In blind Geophilomorpha, the description for indirect mating of *Geophilus flavus* (De Geer, 1778) offers a valuable clue that ultimate legs may be involved in courtship behavior as it starts with rhythmic wipes of the posterior body region on the ground displayed by both sexes (Klingel, 1959). Male and female start to tap the anterior and posterior body region using their antennae. Shortly after, male and female separate, and the male produces a web with a spermatophore that is revisited by the female three to four hours later. As mentioned above, sex-specific pheromones may be involved, conceivably in the impregnation of the web.

Courtship behavior in *Lithobius forficatus* also starts with tapping of the antennae on the ultimate legs and the posterior body (Klingel, 1960b). Both sexes intensively flick their ultimate legs, which lasts for several hours with animals frequently circling each other while tapping the mates’ ultimate legs. Sometimes, they separate and join again, and resume tapping the ultimate legs. The female, still tapping, then follows the male, which may last for half an hour. Eventually, the spermatophore is placed in a web and the female seizes it with her gonopods. The importance of ultimate legs of scolopendromorphs in mating is well documented and has been recognized as such in detail, also due to the popularity of these animals in the community of amateur and professional centipede enthusiasts. Prior to mating, scolopendromorph males also show a distinct courtship behavior that is remarkably similar to what is known for *S. coleoptrata.* The receptiveness of the female is assessed when the male exposes and shakes the ultimate legs towards the head and antennae of the female (Fig. 9B). Klingel (1960a) and Radl (1993) described courtship behavior and mating in *Scolopendra cingulata* Latreille, 1829. The male starts with antennal contact on the posterior body and the ultimate legs of the female. Female ultimate legs are tapped approximately 15 times per minute. Specimens unwilling to mate display a typical defense behavior, including embracing, grasping, or even occasionally biting each other (Kronmüller, 2013; McMonigle, 2014). In over 80% of experiments, courtship behavior successfully led to mating (Radl, 1993). A receptive female uses her antennae to tap on the posterior end of the male and most importantly on the ultimate legs. Frequently, both animals form a ring with constant tapping of the ultimate legs (Fig. 9B), which leads to repeated lifting and lowering of the males’ ultimate legs. The female then follows the male, or if lost, even is guided by the males’ ultimate legs towards a suitable place for spermatophore transfer (Klingel, 1960a; Schaller, 1962; Radl, 1993; McMonigle, 2014). Klingel (1960a) deduced that potential sexual partners and their receptive status are recognized by the way they react during the tapping. Lewis (2010) also conjectured that the sexually dimorphic shaping of ultimate legs is a key aspect of recognizing potential mates.

## Conclusions and Future Directions

### Arthropod legs and homonymous podomeres

As laid out above, ultimate legs in centipedes are special, both in their morphological and functional complexity. Anatomical data on arthropodia of centipedes are restricted to a few, yet elaborate studies (Manton, 1951; Manton, 1958a; Manton, 1958b; Manton, 1965; Manton, 1973; Manton, 1977). Thus, to date it seems virtually impossible to convincingly answer questions on alignment and homology of centipede legs, and particular podomeres. However, assessing serial homology of homonymous podomeres is also challenging in different higher-order taxa of arthropods. Morphological data gathered so far by various authors have led to conflicting conclusions. For example, Bitsch (2001) homologized the hexapod coxa with the basis of biramous malacostracan limbs, whereas the hexapod subcoxa might correspond to the malacostracan coxa (see also Bretfeld, 1963; Larink, 1969; Machida, 1981; Ikeda & Machida, 1998; Bäcker, Fanenbruck & Wägele, 2008; Ferrari & Ivanenko, 2009). The homology of the hexapod subcoxa and crustacean coxa was also supported by identical innervation patters of excitatory motoneurons in crayfish and locusts (Wiens & Wolf, 1993). Thus, the hexapod ‘coxa’ is probably not homologous with the malacostracan ‘coxa’. In myriapods, however, the situation is even more ambiguous as their walking and ultimate legs may include a high number of elements, whose identities are still unclear or insufficiently analyzed (see also Shear, Jeram & Selden, 1998). A famous example in Myriapoda is the subdivision of the diplopod walking leg into subcoxa, coxa, trochanter, prefemur, femur, postfemur, tibia, tarsus, and claw. At least for few diplopod species it is known that coxa and trochanter cannot be compared with homonymous segments of other arthropods as they are derived by fragmentation of a single segment. The same applies to the diplopod femur and postfemur (Manton, 1958b). Likewise, in the centipede *Lithobius forficatus*, trochanter and prefemur are considered as derivatives of a single podomere. While the prefemur (also called trochanter 2; Snodgrass, 1935; Rilling, 1968) is equipped with intrinsic muscles, the trochanter is not (Manton, 1977), and it is still unclear whether this is also the case in Geophilomorpha (where the trochanter is movable). As described above, conflicting denominations of podomeres in arthropodia of Scolopendromorpha and Geophilomorpha refer to post-femoral elements. Only detailed anatomical studies on intrinsic musculature can clarify the origin of elements described as pretibia and tibia, as well as tarsalia. Certainly, the terminology of specific podomeres (for instance ‘prefemur’, ‘postfemur’, and ‘pretibia’) needs to be reconsidered within a given taxon. Homologization of homonymous elements across arthropod taxa appears difficult if not inappropriate due to divergent evolutionary modifications in e.g., Chilopoda and Diplopoda. Additional developmental data will prove crucial in this context and will facilitate our understanding of podomere identity and homology.

### Why study ultimate legs in centipedes?

Ultimate legs of centipedes are a promising example to study and trace functional and constructional aspects of leg modification. Amongst arthropods, this is a rare case study as, in clear contrast to other modified arthropodia, it is always the posterior-most pair of legs in centipedes that displays morphological *and* functional disparity. Thickening, widening and reinforcement with a multitude of cuticular protuberances and glandular systems suggest a role in both attack, as well as defense. Remarkably, in blind scolopendromorphs such as the genera *Cryptops*, *Theatops*, *Tidops*, and *Newportia*, ultimate leg modification is eye-catching. These taxa constitute a monophyletic taxon (Vahtera, Edgecombe & Giribet, 2012) living in caves, crevices or in the soil, and represent a formidable example of structural diversification in rather similar environments. The function of (mostly) ultimate leg associated coxal organs is insufficiently understood, but morphological, behavioral and ecological data indicate a dual function of water uptake and pheromone secretion. Sexually dimorphic characteristics, as well as behavioral observations indicate a pivotal role in intraspecific communication, mate finding, as well as courtship behavior. Yet, without further ethological investigations on movements and utilizations of ultimate legs most conclusions on their functional diversity merely are a matter of conjecture.

In species with multi-annulated tarsi, the predominant function is most likely a sensory one. In terms of sensory biology, this is a fascinating example to explore common functional and constructional principles that led to the evolution of similarly organized sensory organs and presumptive associated processing centers in the nervous system (compare Schachtner, Schmidt & Homberg, 2005; Sombke et al., 2012). Although the antennae (first antennae, also called antennules) of Mandibulata as specialized sensory appendages at the anterior pole possess a unique shape different to all other post-antennular arthropodia (Scholtz & Edgecombe, 2005; Scholtz & Edgecombe, 2006), all appendages of an arthropod’s body are regarded as serially homologous (Boxshall, 2013). The evolutionary pathways to be explored however, likely are the results of convergence. Besides the antennules, legs with a primary sensory function are known from various chelicerates (e.g., Amblypygi, Solifugae, Palpigradi, and Uropygi), but also from Protura, a crypticgroup of wingless hexapods lacking antennae (Dallai & Nosek, 1981; Foelix & Hebets, 2001; Foelix, 2010; Strausfeld, 2012). Interestingly, transformations of arthropodia into sensory appendages at the posterior pole of the body similar to Chilopoda apply to cercophoran Hexapoda (Diplura and Insecta), namely by the evolution of cerci (e.g., Heusslein & Gnatzy, 1987). As for this case, drawing plausible evolutionary scenarios is hampered by the fact that it can only be assumed if the last common ancestor of Cercophora (Kukalová-Peck, 1991; Misof et al., 2014) derived its cerci from former walking legs (see also Kukalová-Peck, 1997). To understand the sensory capacity of centipede ultimate legs in comparison with antennae (compare Müller et al., 2011; Sombke & Rosenberg, 2016), it is necessary to obtain broad knowledge with respect to typological diversity and distribution of cuticular sensilla, as well as innervation patterns covering the arrangement of afferent nerves, and the organization of processing neuropils in the ventral nerve cord.

We advocate that studying centipede ultimate legs is not only essential and worth in filling pre-existing gaps of knowledge in descriptive morphology and sensory biology, but also provides an interesting opportunity to explore evolutionary pathways of leg transformation at the interface of functional and constructional constraints shaping an arthropodium.

## Additional Information and Declarations

### Data Availability

The following information was supplied regarding data availability: Original data are included as images in this contribution.

## References

[ref-1] Attems CG (1930). Das tierreich. Eine zusammenstellung und kennzeichnung der rezenten tierformen. Myriapoda. 2. Scolopendromorpha.

[ref-2] Bäcker H, Fanenbruck M, Wägele JW (2008). A forgotten homology supporting the monophyly of Tracheata: the subcoxa of insects and myriapods re-visited. Zoologischer Anzeiger.

[ref-3] Barber AD (2009). Centipedes.

[ref-4] Bitsch J (2001). The hexapod appendage: basic structures, development and origin. Annales de la Société Entomologique De France.

[ref-5] Bonato L, Edgecombe G, Lewis J, Minelli A, Pereira L, Shelley R, Zapparoli M (2010). A common terminology for the external anatomy of centipedes (Chilopoda). ZooKeys.

[ref-6] Boxshall GA, Fortey RA, Thomas RH (1997). Comparative limb morphology in major crustacean groups: the coxa-basis joint in postmandibular limbs. Arthropod relationships.

[ref-7] Boxshall GA (2004). The evolution of arthropod limbs. Biological Reviews.

[ref-8] Boxshall G, Minelli A, Boxshall G, Fusco G (2013). Arthropod limbs and their development. Arthropod biology and evolution.

[ref-9] Brena C (2014). The embryoid development of *Strigamia maritima* and its bearing on post-embryonic segmentation of geophilomorph centipedes. Frontiers in Zoology.

[ref-10] Brena C, Akam M (2012). The embryonic development of the centipede *Strigamia maritima*. Developmental Biology.

[ref-11] Bretfeld G (1963). Zur anatomie und embryologie der rumpfmuskulatur und der abdominalen anhänge der collembolen. Zoologische Jahrbücher Abteilung für Anatomie und Ontogenie der Tiere.

[ref-12] Brölemann HW (1912). The Myriapoda in the Australian museum. Part I.—Chilopoda. Records of the Australian Museum.

[ref-13] Brölemann HW (1930). Éléments d’une Faune des Myriapodes de France - Chilopodes.

[ref-14] Bücherl W, Bücherl W, Buckley EE (1971). Venomous chilopods or centipedes. Venomous animals and their venom 3.

[ref-15] Carpenter CC, Gillingham JC (1984). Giant centipede (*Scolopendra alternans*) attacks marine toad (*Bufo marinus*). Caribbean Journal of Science.

[ref-16] Chagas-Júnior A (2011). A review of the centipede genus *Tidops* Chamberlin (Scolopendromorpha, Scolopocryptopidae, Newportiinae). International Journal of Myriapodology.

[ref-17] Chagas-Júnior A (2012). The centipede genus *Otostigmus* Porat in Brazil: Description of three new species from the Atlantic Forest; a summary and an identification key to the Brazilian species of this genus (Chilopoda, Scolopendromorpha, Scolopendridae, Otostigminae). Zootaxa.

[ref-18] Chagas-Júnior A, Bichuette ME (2015). A new species of *Scolopocryptops* Newport: a troglobitic scolopocryptopine centipede from a remarkable siliciclastic area of eastern Brazil (Scolopendromorpha, Scolopocryptopidae, Scolopocryptopinae). ZooKeys.

[ref-19] Chagas-Júnior A, Edgecombe GD, Minelli A (2008). Variability in trunk segmentation in the centipede order Scolopendromorpha: a remarkable new species of *Scolopendropsis* Brandt (Chilopoda: Scolopendridae) from Brazil. Zootaxa.

[ref-20] Cloudsley-Thompson JL Spiders, scorpions, centipedes and mites.

[ref-21] Dallai R, Nosek J (1981). Ultrastructure of sensillum t1 on the foretarsus of *Acerentomon majus* Berlese (Protura: Acerentomidae). International Journal of Insect Morphology and Embryology.

[ref-22] Dathe HH (2003). Lehrbuch der speziellen zoologie. Wirbellose tiere. 5. Teil insecta.

[ref-23] Di ZY, Cao ZJ, Wu YL, Yin SJ, Edgecombe GD, Li WX (2010). Discovery of the centipede family Plutoniumidae (Chilopoda) in Asia: a new species of Theatops from China, and the taxonomic value of spiracle distributions in Scolopendromorpha. Zootaxa.

[ref-24] Dobroruka LJ (1961). Die hundertfüssler (Chilopoda).

[ref-25] Dugon MM, Arthur W (2012). Comparative studies on the structure and development of the venom-delivery system of centipedes, and a hypothesis on the origin of this evolutionary novelty. Evolution and Development.

[ref-26] Dugon MM, Black A, Arthur W (2012). Variation and specialisation of the forcipular apparatus of centipedes (Arthropoda: Chilopoda): a comparative morphometric and microscopic investigation of an evolutionary novelty. Arthropod Structure & Development.

[ref-27] Eason EH (1964). Centipedes of the British Isles.

[ref-28] Eason EH (1973). The type specimens and identity of the species described in the genus *Lithobius* by R. I. Pocock from 1890 to 1901 (Chilopoda, Lithobiomorpha). Bulletin of the British Museum (Natural History).

[ref-29] Edgecombe GD, Minelli A (2011a). Chilopoda—fossil history. Treatise on zoology-anatomy, taxonomy, biology. The Myriapoda.

[ref-30] Edgecombe GD, Minelli A (2011b). Chilopoda—phylogeny. Treatise on zoology-anatomy, taxonomy, biology. The Myriapoda.

[ref-31] Edgecombe GD, Minelli A (2011c). Order Scutigeromorpha. Treatise on zoology-anatomy, taxonomy, biology. The Myriapoda.

[ref-32] Edgecombe GD, Barrow L (2007). A new genus of scutigerid centipedes (Chilopoda) from Western Australia, with new characters for morphological phylogenetics of Scutigeromorpha. Zootaxa.

[ref-33] Edgecombe GD, Bonato L (2011). Order Scolopendromorpha. Treatise on zoology-anatomy, taxonomy, biology. The Myriapoda.

[ref-34] Edgecombe GD, Giribet G (2006). A century later—a total evidence re-evaluation of the phylogeny of scutigeromorph centipedes (Myriapoda: Chilopoda). Invertebrate Systematics.

[ref-35] Edgecombe GD, Giribet G (2007). Evolutionary biology of centipedes (Myriapoda: Chilopoda). Annual Review of Entomology.

[ref-36] Ernst A, Rosenberg J (2003). Structure and distribution of sensilla coeloconica on the maxillipedes of Chilopoda. African Invertebrates.

[ref-37] Fernández R, Edgecombe GD, Giribet G (2016). Exploring phylogenomic relationships within Myriapoda: should high matrix occupancy be the goal?. Systematic Biology.

[ref-38] Ferrari FD, Ivanenko VN (2009). Remarks on the “Subcoxa” hypothesis from Backer et al. (2008). Zoologischer Anzeiger—a Journal of Comparative Zoology.

[ref-39] Foelix RF (2010). Biology of spiders.

[ref-40] Foelix R, Hebets E (2001). Sensory biology of whip spiders (Arachnida, Amblypygi). Andrias.

[ref-41] Gerstäcker A (1854). Ueber eine neue Myriapoden-und Isopoden-Gattung. Entomologische Zeitung Stettin.

[ref-42] Gowri N, Nageswaran R (1981). An investigation on the structure and function of the sense organs in the anal legs of *Geophilus subterraneous* (Chilopoda: Myriapoda). The Indian Zoologist.

[ref-43] Gröhn C (2015). Einschlüsse im baltischen bernstein.

[ref-44] Haake W (1885). Schildasseln auf der fliegenjagd. Der Zoologische Garten.

[ref-45] Haake W (1886). Beobachtungen über lebensweise und gliedmassenbau der schildassel, *Scutigera smithii* (Newp.). Der Zoologische Garten.

[ref-46] Haug JT, Haug C, Schweigert G, Sombke A (2014). The evolution of centipede venom claws—open questions and possible answers. Arthropod Structure & Developmen.

[ref-47] Heusslein R, Gnatzy W (1987). Central projections of campaniform sensilla on the cerci of crickets and cockroaches. Cell and Tissue Research.

[ref-48] Heymons R (1901). Die entwicklungsgeschichte der Scolopender. Zoologica.

[ref-49] Ikeda Y, Machida R (1998). Embryogenesis of the dipluran *Lepidocampa weberi* Oudemans (Hexapoda, Diplura, Campodeidae): external morphology. Journal of Morphology.

[ref-50] Iorio E (2003). La fonction stridulatoire des Scolopendres du genre *Alipes* Imhoff, 1854 (Chilopoda, Scolopendromorpha, Scolopendridae, Otostigminae). Bulletin de Phyllie.

[ref-51] Jangi BS (1961). The skeleto-musculatur mechanism of the so-called anal legs in the centipede *Scolopendra amazonica* (Scolopendridae). Annals of the Entomological Society of America.

[ref-52] Jangi BS (1964). Sensory physiology of the anal legs of centipedes. Current Science.

[ref-53] Kaestner A (1963). Lehrbuch der speziellen zoologie.

[ref-54] Karlinski J (1883). O gruczolach jadowych w szczekonozach drewniakow (Lithobiidae). Kosmos.

[ref-55] Keil T (1975). Die Antennensinnes- und Hautdrüsenorgane von *Lithobius forficatus* L. Eine licht- und elektronenmikroskopische Untersuchung. Inaugural-Dissertation Thesis.

[ref-56] Klass KD, Kristensen NP (2001). The ground plan and affinities of hexapods: recent progress and open problems. Annales de la Société Entomologique de France.

[ref-57] Klingel H (1959). Indirekte Spermatophoren übertragung bei Geophiliden (Hundertfüssler, Chilopoda). Naturwissenschaften.

[ref-58] Klingel H (1960a). Vergleichende verhaltensbiologie der Chilopoden *Scutigera coleoptrata* L. (“Spinnenassel”) und *Scolopendra cingulata* Latreille (Skolopender). Zeitschrift für Tierpsychologie.

[ref-59] Klingel H (1960b). Die paarung des *Lithobius forficatus*. Zoologischer Anzeiger.

[ref-60] Klingel H (1962). Das Paarungsverhalten des malaiischen Höhlentausendfusses *Thereuopoda decipiens cavernicola* Verhoeff (Scutigeromorpha, Chilopoda). Zoologischer Anzeiger.

[ref-61] Koch CL, Berendt GC (1854). Die im Bernstein befindlichen crustaceen, myriapoden, arachniden und apteren der vorwelt.

[ref-62] Koehl MAR, Breithaupt T, Thiel M (2011). Hydrodynamics of sniffing by crustaceans. Chemical communication in crustaceans.

[ref-63] Kronmüller C (2013). Hundertfüßer.

[ref-64] Kronmüller C, Lewis JGJ (2015). On the function of the ultimate legs of some Scolopendridae (Chilopoda, Scolopendromorpha). ZooKeys.

[ref-65] Kukalová-Peck J, Naumann ID (1991). Fossil history and the evolution of hexapod structures. The insects of Australia: a textbook for students and researchers.

[ref-66] Kukalová-Peck J, Fortey RA, Thomas RH (1997). Arthropod phylogeny and basal morphological structures. Arthropod relationships.

[ref-67] Larink O (1969). Zur entwicklungsgeschichte von *Petrobius brevistyli* (Thysanura, Insecta). Helgoländer Wissenschaftliche Meeresuntersuchungen.

[ref-68] Latzel R (1880). Die Myriopoden der Österreichisch-Ungarischen Monarchie. Erste Hälfte: Die Chilopoden.

[ref-69] Lawrence RF (1953). The biology of the cryptic fauna of forests with special reference to the indigenous forest of South Africa.

[ref-70] Lawrence RF (1975). A new subspecies of wing-footed centipedes, *Alipes* Imhoff, from Rhodesia (Chilopoda: Scolopendromorpha). Arnoldia.

[ref-71] Lewis JGE (1981). The biology of centipedes.

[ref-72] Lewis JGE, Ellis WN, Jeekel CAW, Pieters FFJM (1985). Possible isolation mechanisms in some scolopendrid centipedes (Chilopoda, Scolopendridae).

[ref-73] Lewis JGE (2010). On the function of the ultimate legs of *Cryptops* and *Theatops* (Chilopoda, Scolopendromorpha). International Journal of Myriapodology.

[ref-74] Littlewood PMH (1983). Fine structure and function of the coxal glands of lithobiomorph centipedes: *Lithobius forficatus* L. and *L. crassipes* L. Koch (Chilopoda, Lithobiidae). Journal of Morphology.

[ref-75] Littlewood PMH (1988). The chemosensory behaviour of *Lithobius forficatus* (Myriapoda: Chilopoda). 2. Bioassay and chemistry of the coxal pheromone. Journal of Zoology: Proceedings of the Zoological Society of London.

[ref-76] Littlewood PMH (1991a). Chilopod coxal organs: morphological considerations with reference to function. Journal of Zoology: Proceedings of the Zoological Society of London.

[ref-77] Littlewood PMH (1991b). The water relations of *Lithobius forficatus* and the role of the coxal organs (Myriapoda: Chilopoda). Journal of Zoology.

[ref-78] Littlewood PMH, Blower JG (1987). The chemosensory behaviour of *Lithobius forficatus*. 1. Evidence for a pheromone released by the coxal organs (Myriapoda: Chilopoda). Journal of Zoology: Proceedings of the Zoological Society of London.

[ref-79] Ma H, Song D, Zhu M (2007). Review of *Cermatobius* Haase, 1885 (Chilopoda: Henicopidae) of China and neotype designation for *Cermatobius longicornis* (Takakuwa, 1939). Zootaxa.

[ref-80] Machida R (1981). External features of embryonic development of a jumping bristletail, *Pedetonus unimaculatus* Machida (Insecta, Thysanura, Machilidae). Journal of Morphology.

[ref-81] Manton SM (1951). The evolution of arthropod locomotory mechanism. Part 3. The locomotion of Chilopoda and Pauropoda. Journal of the Linnean Society of London/Zoology.

[ref-82] Manton SM (1958a). Habits of life and evolution of body design in Arthropoda. Journal of the Linnean Society of London.

[ref-83] Manton SM (1958b). The evolution of arthropodan locomotory mechanisms. Part 6. Habits and evolution of the Lysiopetaloidea (Diplopoda), some principles of the leg design in Diplopoda and Chilopoda, and limb structure in Diplopoda. Journal of the Linnean Society of London.

[ref-84] Manton SM (1965). The evolution of arthropodan locomotory mechanisms. Part 8. Functional requirements and body design in Chilopoda. Journal of the Linnean Society of London.

[ref-85] Manton SM (1973). The evolution of arthropod locomotory mechanisms Part 11. Habits, morphology and evolution of Uniramia (Onychophora, Myriapoda, Hexapoda) and comparison with the Arachnida, together with a functional review of uniramian musculature. Zoological Journal of the Linnean Society.

[ref-86] Manton SM (1977). The Arthropoda. Habits, functional morphology, and evolution.

[ref-87] Manton SM, Gupta AP (1979). Functional morphology and the evolution of the Hexapod classes. Arthropod phylogeny.

[ref-88] Martínez-Muñoz CA, Dolejš P, Kronmüller C (2016). On the true identity of *Scolopendra aztecorum* Verhoeff, 1934 (Chilopoda: Scolopendromorpha: Scolopendridae). Ecologica Montenegrina.

[ref-89] Maruzzo D, Bonato L (2014). Morphology and diversity of the forcipules in *Strigamia centipedes* (Chilopoda, Geophilomorpha). Arthropod Structure & Developmen.

[ref-90] Maruzzo D, Bonato L, Brena C, Fusco G, Minelli A, Koenemann S, Jenner RA (2005). Appendage loss and regeneration in arthropods: a comparative view. Crustacea and arthropod relationships.

[ref-91] McLaughlin PA, Abele LG (1982). Comparative morphology of crustacean appendages. The biology of crustacea.

[ref-92] McMonigle O (2014). Centipedes in captivity—the reproductive biology and husbandry of chilopoda.

[ref-93] Minelli A (2011). Treatise on zoology-anatomy, taxonomy, biology. The Myriapoda.

[ref-94] Minelli A, Koch M, Minelli A (2011). Chilopoda—general morphology. Treatise on zoology-anatomy, taxonomy, biology. The Myriapoda.

[ref-95] Minelli A, Sombke A, Minelli A (2011). Chilopoda—development. Treatise on zoology-anatomy, taxonomy, biology. The Myriapoda.

[ref-96] Misioch M (1978). Variations in characters in some geophilid chilopods. Abhandlungen und Verhandlungen Naturwissenschaftlicher Verein Hamburg.

[ref-97] Misof B, Liu S, Meusemann K, Peters RS, Donath A, Mayer C, Frandsen PB, Ware J, Flouri T, Beutel RG, Niehuis O, Petersen M, Izquierdo-Carrasco F, Wappler T, Rust J, Aberer AJ, Aspöck U, Aspöck H, Bartel D, Blanke A, Berger S, Böhm A, Buckley TR, Calcott B, Chen J, Friedrich F, Fukui M, Fujita M, Greve C, Grobe P, Gu S, Huang Y, Jermiin LS, Kawahara AY, Krogmann L, Kubiak M, Lanfear R, Letsch H, Li Y, Li Z, Li J, Lu H, Machida R, Mashimo Y, Kapli P, McKenna DD, Meng G, Nakagaki Y, Navarrete-Heredia JL, Ott M, Ou Y, Pass G, Podsiadlowski L, Pohl H, Von Reumont BM, Schütte K, Sekiya K, Shimizu S, Slipinski A, Stamatakis A, Song W, Su X, Szucsich NU, Tan M, Tan X, Tang M, Tang J, Timelthaler G, Tomizuka S, Trautwein M, Tong X, Uchifune T, Walzl MG, Wiegmann BM, Wilbrandt J, Wipfler B, Wong TKF, Wu Q, Wu G, Xie Y, Yang S, Yang Q, Yeates DK, Yoshizawa K, Zhang Q, Zhang R, Zhang W, Zhang Y, Zhao J, Zhou C, Zhou L, Ziesmann T, Zou S, Li Y, Xu X, Zhang Y, Yang H, Wang J, Wang J, Kjer KM, Zhou X (2014). Phylogenomics resolves the timing and pattern of insect evolution. Science.

[ref-98] Molinari J, Gutiérrez EE, De Ascençccão AA, Nassar JM, Arends A, Márquez RJ (2005). Predation by giant centipedes, *Scolopendra gigantea*, on three species of bats in a Venezuelan cave. Caribbean Journal of Science.

[ref-99] Müller CHG, Rosenberg J (2009). Morphology is still an indispensable discipline in zoology: facts and gaps from Chilopoda. Soil Organisms.

[ref-100] Müller CHG, Sombke A, Hilken G, Rosenberg J, Minelli A (2011). Chilopoda—sense organs. Treatise on zoology-anatomy, taxonomy, biology. The Myriapoda.

[ref-101] Panic J (1963). Das verhalten von ameisen gegenüber bodenbewohnenden kleinarthropoden. Pedobiologia.

[ref-102] Pocock RI (1896). Descrition of a new species of the leaf-footed centipede (*Alipes*) from Nyasaland, together with notes upon the previously described species of the genus. Journal of Natural History Series 6.

[ref-103] Radl RC (1993). Über lebenszyklus, fortpflanzung und brutpflege des hundertfüßers *Scolopendra cingulata* (Chilopoda, Scolopendromorpha). Dissertation Thesis.

[ref-104] Rajulu GS (1970). A study on the chemo- and mechanoreceptors in the last pair of legs of a geophilomorph centipede *Himantarium samuelraji* Rajulu (Chilopoda: Myriapoda). Monitore Zoologico Italiano.

[ref-105] Rilling G (1968). Lithobius forficatus. Grosses Zoologisches Praktikum 13b.

[ref-106] Rosenberg J (1982). Coxal organs in Geophilomorpha (Chilopoda). Organization and Fine Structure of the Transporting Epithelium. Zoomorphology.

[ref-107] Rosenberg J (1983a). Coxal organs in Scolopendromorpha (Chilopoda): Topography, organization, fine structure and signification in centipedes. Zoologische Jahrbücher Abteilung für Anatomie und Ontogenie der Tier.

[ref-108] Rosenberg J (1983b). Coxal organs of *Lithobius forficatus* (Myriapoda, Chilopoda): fine structural investigation with special reference to the transport epithelium. Cell & Tissue Research.

[ref-109] Rosenberg J (1988a). Bestimmungsschlüssel für mitteleuropäische Erdläufer (Geophilomorpha) anhand der Coxalporen. Acta Biologica Benrodis.

[ref-110] Rosenberg J (1988b). Hundertfüßler unter dem mikroskop: ein wichtiges bestimmungsmerkmal: coxalporen bei erdläufern. Mikrokosmos.

[ref-111] Rosenberg J (2009). Die Hundertfüßer.

[ref-112] Rosenberg J, Bajorat KH (1984). Einfluß der coxalorgane von *Lithobius forficatus* L. (Chilopoda) auf die sorption von wasserdampf. Zoologische Jahrbücher Allgemeine Zoologie und Physiologie der Tier.

[ref-113] Rosenberg J, Brenner M, Greven H (2004). Putzverhalten und trinken bei *Scutigera coleoptrata* L. (Chilopoda, Scutigeromorpha). Entomologie Heute.

[ref-114] Rosenberg J, Brenner M, Greven H (2005). Preening and drinking in *Scutigera coleoptrata* (Chilopoda: Scutigeromorpha) (Video-film).

[ref-115] Rosenberg J, Müller CHG (2009). Morphology in Chilopoda—a survey. Soil Organisms.

[ref-116] Rosenberg J, Müller CHG, Hilken G (2006). Ultrastructural organization of the anal organs in the anal capsule of *Craterostigmus tasmanianus* Pocock, 1902 (Chilopoda, Craterostigmomorpha). Journal of Morphology.

[ref-117] Rosenberg J, Müller CHG, Hilken G, Minelli A (2011a). Chilopoda–Integument and associated organs. The Myriapoda.

[ref-118] Rosenberg J, Müller CHG, Hilken G, Minelli A (2011b). Coxal and anal organs. Treatise on zoology-anatomy, taxonomy, biology. The myriapoda.

[ref-119] Ruppert A (2015). The sound of a flag tail centipede. https://www.youtube.com/watch?v=I0Q442yswr8.

[ref-120] Schachtner J, Schmidt M, Homberg U (2005). Organization and evolutionary trends of primary olfactory brain centers in Tetraconata (Crustacea+Hexapoda). Arthropod Structure & Development.

[ref-121] Schaller F (1962). Die unterwelt des tierreiches.

[ref-122] Schileyko A (2009). *Ectonocryptopoides sandrops*—a new scolopendromorph centipede from Belize. Soil Organisms.

[ref-123] Schileyko AA (2013). A new species of *Newportia* Gervais, 1847 from Puerto Rico, with a revised key to the species of the genus (Chilopoda, Scolopendromorpha, Scolopocryptopidae). ZooKeys.

[ref-124] Schileyko A, Minelli A (1998). On the genus *Newportia* Gervais, 1847 (Chilopoda: Scolopendromorpha: Newportiidae). Arthropoda Selecta.

[ref-125] Scholtz G, Edgecombe GD, Koenemann S, Jenner RA (2005). Heads, Hox and the phylogentic position of trilobites. Crustacea and arthropod relationships.

[ref-126] Scholtz G, Edgecombe GD (2006). The evolution of arthropod heads: reconciling morphological, developmental and palaeontological evidence. Development Genes and Evolution.

[ref-127] Schram FR (1986). Crustacea.

[ref-128] Shear WA, Jeram AJ, Selden PA (1998). Centipede legs (Arthropoda, Chilopoda, Scutigeromorpha) from the Silurian and Devonian of Britain and the Devonian of North America. American Museum Novitates.

[ref-129] Shelley RM (1990). The centipede *Theatops posticus* (Say.) (Scolopendromorpha: Cryptodidae) in the Southwestern United States and Mexico. Canadian Journal of Zoology.

[ref-130] Shelley RM (1997). The holarctic centipede subfamily Plutoniuminae (Chilopoda: Scolopendromorpha: Cryptopidae) (Nomen correctum ex subfamily Plutoniinae Bollman, 1893). Brimleyana.

[ref-131] Shelley RM (2002). A synopsis of the North American centipedes of the order Scolopendromorpha (Chilopoda).

[ref-132] Shelley RM, Mercurio R (2005). *Ectonocryptoides quadrimeropus*, a new centipede genus and species from Jalisco, Mexico; proposal of Ectonocryptopinae, analysis of subfamilial relationships, and a key to subfamilies and genera of the Scolopocryptopidae (Scolopendromorpha). Zootaxa.

[ref-133] Shelley RM, Mercurio R (2008). Redescription and illustrations of the centipede, *Ectonocryptops kraepelini* Crabill, 1977 (Scolopendromorpha: Scolopocryptopidae: Ectonocryptopinae). Zootaxa.

[ref-134] Simon HR (1964). Zum abwehrverhalten von *Lithobius forficatus* (Myriapoda, Chilopoda). Entomologische Zeitschrif.

[ref-135] Siriwut W, Edgecombe GD, Sutcharit C, Panha S (2014). Brooding behaviour of the centipede *Otostigmus spinosus* Porat, 1876 (Chilopoda: Scolopendromorpha: Scolopendridae) and its morphological variability in Thailand. Raffles Bulletin of Zoology.

[ref-136] Siriwut W, Edgecombe G, Sutcharit C, Tongkerd P, Panha S (2016). A taxonomic review of the centipede genus *Scolopendra* Linnaeus, 1758 (Scolopendromorpha, Scolopendridae) in mainland Southeast Asia, with description of a new species from Laos. ZooKeys.

[ref-137] Skovmand O, Enghoff H (1980). Stridulation in *Alipes grandidieri* (Lucas), a Scolopendromorph Centipede. Vidensk. Meddr. dansk naturh. Foren.

[ref-138] Snodgrass RE (1935). Principles of insect morphology.

[ref-139] Snodgrass RE (1952). A textbook of arthropod anatomy.

[ref-140] Sombke A, Edgecombe GD (2014). Morphology and evolution of Myriapoda. Arthropod Structure & Developmen.

[ref-141] Sombke A, Lipke E, Kenning M, Müller CH, Hansson BS, Harzsch S (2012). Comparative analysis of deutocerebral neuropils in Chilopoda (Myriapoda): implications for the evolution of the arthropod olfactory system and support for the Mandibulata concept. BMC Neuroscience.

[ref-142] Sombke A, Rosenberg J, Schmidt-Rhaesa A, Harzsch S, Purschke G (2016). Myriapoda. Structure and evolution of invertebrate nervous systems.

[ref-143] Sombke A, Rosenberg J, Hilken G, Westermann M, Ernst A (2011). The source of chilopod sensory information: external structure and distribution of antennal sensilla in *Scutigera coleoptrata* (Chilopoda, Scutigeromorpha). Journal of Morphology.

[ref-144] Strausfeld NJ (2012). Arthropod brains. Evolution, functional elegance, and historical significance.

[ref-145] Undheim E, Fry B, King G (2015). Centipede venom: recent discoveries and current state of knowledge. Toxins.

[ref-146] Undheim EAB, Jones A, Clauser KR, Holland JW, Pineda SS, King GF, Fry BG (2014). Clawing through evolution: toxin diversification and convergence in the ancient lineage chilopoda (Centipedes). Molecular Biology and Evolution.

[ref-147] Undheim EAB, King GF (2011). On the venom system of centipedes (Chilopoda), a neglected group of venomous animals. Toxicon.

[ref-148] UnicoCelula (2012). *Theatops* sp. feeding with terminal legs. https://www.youtube.com/watch?v=i1sgW_fj2T4.

[ref-149] Vahtera V, Edgecombe GD, Giribet G (2012). Evolution of blindness in scolopendromorph centipedes (Chilopoda: Scolopendromorpha): insight from an expanded sampling of molecular data. Cladistics.

[ref-150] Vahtera V, Edgecombe GD, Giribet G (2013). Phylogenetics of scolopendromorph centipedes: can denser taxon sampling improve an artificial classification?. Invertebrate Systematics.

[ref-151] Verhoeff C (1898). Beiträge zur kenntniss paläarktischer Myriopoden. VI. Aufsatz: über paläarktische Geophiliden. Archiv für Naturgeschichte.

[ref-152] Verhoeff KW (1902). Abteilung Gliederfüssler: arthropoda klasse chilopoda. Bronn’s klassen und ordnungen des tier-Reichs.

[ref-153] Verhoeff KW (1903). Über Tracheaten-Beine. Vierter und fünfter Aufsatz: Chilopoda und Hexapoda. Vierter Aufsatz: Chilopoden-Beine und Muskelgesetze. Nova acta Academiae Caesareae Leopoldino-Carolinae Germanicae Naturae Curiosorum.

[ref-154] Verhoeff KW (1905). Über die entwicklungsstufen der steinläufer, Lithobiiden, und beiträge zur kenntnis der Chilopoden. Zoologische Jahrbücher: Zeitschrift für Systematik, Geographie und Biologie der Tiere.

[ref-155] Verhoeff KW (1935). Die tierwelt mitteleuropas. Diplopoda, symphyla, pauropoda, chilopoda.

[ref-156] Waldrop LD, Koehl MAR (2016). Do terrestrial hermit crabs sniff? Air flow and odorant capture by flicking antennules. Journal of the Royal Society Interface.

[ref-157] Walossek D, Müller KJ, Fortey RA, Thomas RH (1997). Cambrian Orsten-type arthropods and the phylogeny of Crustacea. Arthropod relationships.

[ref-158] Waloszek D, Chen J, Maas A, Wang X (2005). Early Cambrian arthropods—new insights into arthropod head and structural evolution. Arthropod Structure & Development.

[ref-159] Weygoldt P (1996). Evolutionary morphology of whip spiders: towards a phylogenetic system (Chelicerata: Arachnida: Amblypygi). Journal of Zoological Systematics and Evolutionary Research.

[ref-160] Wiens TJ, Wolf H (1993). The inhibitory motoneurons of crayfish thoracic limbs: identification, structures, and homology with insect common inhibitors. Journal of Comparative Neurology.

[ref-161] Willem V (1897). Les glandes filières (coxales) des Lithobies. Annales de la Société Entomologique de Belgique.

[ref-162] Wolf H, Harzsch S (2002). Evolution of the arthropod neuromuscular system. 1. Arrangement of muscles and innervation in the walking legs of a scorpion: *Vaejovis spinigerus* (Wood,1863) Vaejovidae, Scorpiones, Arachnida. Arthropod Structure & Development.

[ref-163] Würmli M, Blower JG (1974). Systematic criteria in the Scutigeromorpha. Symposia of the zoological society of London.

[ref-164] Zapparoli M, Edgecombe GD, Minelli A (2011). Order Lithobiomorha. Treatise on zoology-anatomy, taxonomy, biology. The Myriapoda.

